# Neuroplasticity-informed hierarchical and uncertainty-aware modeling of secondary-school english learning

**DOI:** 10.3389/fnmol.2026.1912397

**Published:** 2026-10-29

**Authors:** Han Wang, Mohd Shahril Nizam Shaharom, Hai-Leng Chin, Yuan He

**Affiliations:** Faculty of Education, Department of Curriculum and Instructional Technology, University of Malaya, Kuala Lumpur, Malaysia

**Keywords:** adaptive pedagogy, classroom learning, high school English, neuroplasticity, uncertainty propagation

## Abstract

**Introduction:**

This study develops a neuroplasticity-informed computational framework for modeling heterogeneous English-learning outcomes in secondary-school educational settings. Rather than treating neuroplasticity as a directly observable neural quantity, the study uses it as a theoretical perspective for conceptualizing learning-related behavior as adaptive, experience-sensitive, and heterogeneous across learners and contexts.

**Methods:**

We propose HierarchicalFactorizedUncertainty, a unified predictive framework that integrates multi-level hierarchical representation learning, sparse low-rank learning–context interaction modeling, and aleatoric–epistemic uncertainty modeling. The Hierarchical Composition Mapper learns structured representations from learner–language evidence, while the Factorized Relation Projector models compact associations between latent representations and recorded task or contextual information. The Uncertainty Propagation Resolver represents observation-dependent predictive variability and model uncertainty using heteroscedastic prediction and stochastic model inference. Sparse low-rank regularization further suppresses weak or redundant interaction factors and improves representation compactness. The framework is evaluated on four complementary learner corpora—CSEE, ELLIPSE, SEEFLEX, and LOCSE—covering written and spoken English performance, heterogeneous task or contextual conditions, and both cross-sectional and longitudinal observations.

**Results and discussion:**

Under controlled data partitions and repeated training runs, Hierarchical Factorized Uncertainty achieves the best mean performance across all reported prediction metrics relative to conventional statistical, recurrent, and pretrained Transformer baselines, although the statistical strength of the improvements varies by dataset and metric. Ablation analyses show that removing the Hierarchical Composition Mapper produces the largest mean deterioration among the architecture- and regularization-related ablations across all four datasets. The Factorized Relation Projector provides an additional consistent predictive contribution, with a comparatively pronounced effect on SEEFLEX, while the relative contributions of the implemented aleatoric and epistemic uncertainty mechanisms vary across datasets and are strongest when both are retained. These findings support structured, context-sensitive, and uncertainty-aware modeling for observable English-learning outcomes while maintaining a clear distinction between behavioral prediction and direct evidence of neuroplastic change. HierarchicalFactorizedUncertainty is therefore intended as a predictive framework with potential decision-support value for English assessment and teacher review rather than as a causal model of instructional effectiveness or neural restructuring.

## 1 Introduction

Adolescence is characterized by continuing neurocognitive development and substantial individual variation in how learners adapt their behavior to changing goals and environmental demands (Wilbrecht and Davidow, 2024). Recent developmental evidence further indicates that adolescent plasticity is embedded in social and environmental contexts, making learning during this period sensitive to differences in experience and opportunity (Baker et al., 2025). Within second-language (L2) research, neurocognitive studies have shown that language experience and proficiency are associated with changes in patterns of neural recruitment and language processing across the course of learning (van Hell, 2023). Direct empirical claims about neuroplastic change, however, are typically grounded in structural, functional, or electrophysiological measurements rather than behavioral language performance alone (Alghamdi, 2026). Accordingly, the present study adopts neuroplasticity as a theoretical lens for conceptualizing learning-related behavior as adaptive, heterogeneous, and experience-sensitive, while restricting its empirical claims to observable English-learning outcomes and latent computational representations inferred from educational data.

Secondary-school English education provides an informative setting for studying such variability because learner performance is expressed through multiple observable dimensions, including written production, spoken performance, proficiency assessment, and responses to heterogeneous language tasks. Learner-corpus research has increasingly connected authentic L2 production with second-language acquisition, foreign-language teaching, statistical analysis, and natural language processing, while emphasizing the importance of learner diversity, metadata, and ecologically grounded language evidence (Granger, 2024). These resources make it possible to model variation in English performance across learners, tasks, and contexts without assuming that behavioral observations constitute direct measurements of underlying neural states. More broadly, recent educational prediction research has increasingly adopted machine-learning and deep-learning approaches to represent complex relationships among learner characteristics, observed performance, and educational context, while identifying explainability and pedagogical relevance as continuing challenges (Boujmiraz et al., 2026). The English-education specificity of the present problem arises from the learner-language evidence, educational context, and supervised outcomes being modeled rather than from an assumption that the computational architecture is intrinsically English-specific. Across the four corpora, the principal predictive evidence consists of learner-produced English texts or transcribed speech, while prompt, task, session, or measurement-occasion information represents recorded educational context. The supervised targets are externally assigned holistic writing or English-proficiency outcomes. Accordingly, the framework addresses heterogeneity in observable English performance across learners and educational conditions. It does not treat vocabulary knowledge, grammar, reading comprehension, fluency, retention, or error type as separately identified constructs unless such variables are explicitly observed and defined as modeling targets.

Advances in natural language processing have substantially expanded the computational tools available for modeling learner language. Automated essay scoring research has progressed from manually engineered linguistic features toward recurrent architectures and pretrained Transformer representations, but robust generalization across prompts, populations, and assessment conditions remains an open problem (Li and Ng, 2024). Recent surveys of deep-learning-based essay assessment further identify reliable score prediction, interpretability, and the connection between automated assessment and meaningful feedback as persistent challenges (Misgna et al., 2025). These issues are particularly relevant to secondary-school English data, where observations may differ in task type, learner background, proficiency level, corpus size, contextual information, and whether repeated measurements are available. A computational model intended for such settings therefore needs to represent structured heterogeneity while avoiding the assumption that a single latent representation or point prediction can fully characterize the variability present in educational observations.

Predictive uncertainty provides one mechanism for addressing this limitation. Modern uncertainty-aware learning distinguishes aleatoric uncertainty associated with observation-dependent variability from epistemic uncertainty associated with limited model knowledge and finite training evidence (Gawlikowski et al., 2023). This distinction is especially relevant when educational predictions are considered for decision support, because similar point predictions may have different implications when they are accompanied by different levels or sources of predictive uncertainty. At the same time, human-centerd research in learning analytics and artificial intelligence in education has emphasized that reliability, transparency, stakeholder agency, and appropriate human control are essential when computational outputs are incorporated into educational practice (Alfredo et al., 2024). Thus, a useful model for English-learning assessment should not only predict learner performance but also represent relevant contextual relationships and communicate predictive uncertainty without presenting statistical associations as causal instructional effects.

These considerations motivate a methodological gap between existing language-performance prediction systems and the requirements of heterogeneous educational data. Conventional and pretrained scoring models can provide strong representations of learner language, but they do not necessarily offer an explicit mechanism for jointly representing multi-level predictive structure, compact associations with recorded task or contextual variables, sparse interaction patterns, and distinct forms of predictive uncertainty within a unified framework. At the same time, computational representations derived from behavioral language data should remain clearly separated from claims about directly measured cognitive or neural change. Addressing these two requirements-structured predictive modeling and explicit interpretive boundaries is central to developing computational methods that are technically useful while remaining educationally defensible.

To address this gap, we propose HierarchicalFactorizedUncertainty, a neuroplasticity-informed computational framework for predicting observable English-learning outcomes in heterogeneous secondary-school settings. The framework combines a Hierarchical Composition Mapper for multi-level representation learning, a Factorized Relation Projector for compact modeling of associations between latent learning representations and recorded task or contextual information, and an Uncertainty Propagation Resolver for representing aleatoric and epistemic predictive uncertainty. Sparse low-rank regularization is further incorporated to suppress weak or redundant interaction factors and improve representation compactness. The framework is predictive rather than causal: its latent representations summarize patterns inferred from observed learner-language and contextual evidence and are not interpreted as direct measurements of neuroplasticity or neural restructuring.

The empirical evaluation uses four complementary English learner corpora-CSEE, ELLIPSE, SEEFLEX, and LOCSE covering written and spoken English performance, cross-sectional and repeated observations, and heterogeneous task or contextual conditions. The framework is compared with conventional statistical models, a recurrent neural baseline, and pretrained Transformer baselines under controlled data partitions, development-set model selection, repeated training runs, and pairwise statistical testing. Component-level ablations further examine the predictive contributions of hierarchical representation learning, factorized learning-context interaction modeling, sparse regularization, and the implemented aleatoric and epistemic uncertainty mechanisms.

We summarize the main contributions as follows:

- We formulate secondary-school English-learning prediction as a neuroplasticity-informed but empirically bounded problem, explicitly distinguishing observable learner-language and educational evidence from latent computational representations while avoiding unsupported claims of direct neural measurement or causal instructional effects.
- We develop HierarchicalFactorizedUncertainty, which integrates multi-level hierarchical representation learning, sparse low-rank learning-context interaction modeling, and explicit aleatoric-epistemic uncertainty modeling within a unified predictive framework.
- We conduct a multi-corpus evaluation across written, spoken, cross-sectional, and longitudinal English-learning settings, using controlled data partitions, repeated training runs, statistical comparisons with representative baselines, and systematic component ablations to examine both overall predictive performance and the contributions of the proposed model components.
- We formulate the model outputs as potential decision-support information for secondary-school English assessment and teacher review, combining performance predictions with contextual representation and predictive uncertainty while retaining human oversight and clearly defining the limits of behavioral-data-based inference.

## 2 Related work

### 2.1 Neuroplasticity and second-language development

Neuroplasticity provides an important theoretical perspective for understanding why language-related behavior can remain responsive to experience during development, but direct evidence of neural plasticity requires biological or neurophysiological measurements rather than behavioral performance alone. A systematic review of training-induced plasticity in children and adolescents identified changes in brain activation, structure, microstructure, and connectivity across a range of training paradigms, while also noting substantial variation in study design and the limited use of rigorous control conditions (Tymofiyeva and Gaschler, 2021). This evidence supports the general principle of experience-dependent neural adaptation during youth, but it does not imply that every observed improvement in educational performance constitutes a measurable neural change. Second-language and bilingualism research provides more language-specific evidence for experience-related neural adaptation. A recent systematic review of the developing bilingual brain synthesized 94 studies and reported that bilingual language experience, proficiency, and exposure are associated with developmental differences in language-related and domain-general neural processing (Yeh et al., 2026). A complementary systematic review and meta-analysis covering bilingual participants from infancy to adolescence identified activation across language and executive-control regions, but also emphasized substantial heterogeneity in participant characteristics, task paradigms, and neuroimaging methodology (Leung et al., 2026). These findings indicate that language experience can be associated with differences in neural organization while simultaneously cautioning against assuming a one-to-one correspondence between behavioral language outcomes and specific neural mechanisms. A broader systematic review of bilingualism and domain-general cognition similarly found associations between bilingual experience and functional or structural brain characteristics, while showing that the observed patterns depend on language-control demands, experience, and methodological choices (Tao et al., 2021). Such findings are relevant to educational research because they motivate attention to individual variability and experience-dependent adaptation, but the neural constructs investigated in this literature are not interchangeable with latent variables learned from classroom or learner-corpus data. This distinction is particularly important for computational studies that use writing scores, speaking proficiency, task descriptors, or repeated behavioral observations as empirical inputs. Educational studies also show that theories of cognition can inform language pedagogy without requiring direct neural measurement. A systematic review of 62 empirical studies on cognitive-linguistics-inspired second-language pedagogies found generally positive learning outcomes across a variety of linguistic targets and instructional approaches, while also documenting considerable diversity in pedagogical implementation and research design (Liu and Qin, 2024). These strands of literature motivate the present study's neuroplasticity-informed view of learning as heterogeneous, adaptive, and experience-sensitive. However, they also establish an important boundary for the current work: HierarchicalFactorizedUncertainty models observable English-learning evidence and latent computational representations, rather than directly estimating neural plasticity or biological cognitive restructuring.

### 2.2 Classroom interaction, engagement, and differentiated instruction

English learning takes place within social and instructional environments in which learner engagement, teacher support, task design, and interaction opportunities can vary substantially. A recent review of classroom environment research in EFL education indicates that interpersonal relationships, classroom climate, instructional support, and opportunities for participation are closely associated with behavioral, cognitive, and emotional dimensions of learner engagement (Ye, 2024). These findings support a view of English-learning performance as context-sensitive rather than as an isolated property of an individual learner. Teacher support represents one particularly important dimension of this educational context. A systematic review of second-language education research found that teacher support is associated with student engagement through multiple motivational and affective pathways, while also revealing considerable variation in how both support and engagement are operationalized across studies (Gan and Lim, 2026). This variability is relevant to computational modeling because broad constructs such as “instructional context” should not be treated as homogeneous or assumed to have identical meanings across datasets. Differentiated instruction provides a related educational framework for responding to learner heterogeneity. A systematic review focused specifically on secondary education found generally small-to-moderate positive evidence for differentiated instructional approaches, but also identified substantial methodological variation and insufficient evidence for strong conclusions about the relative effectiveness of specific differentiation strategies (Smale-Jacobse et al., 2019). This literature supports the educational importance of accommodating differences in proficiency and learning needs while cautioning against assuming that one instructional configuration will be optimal for every learner or classroom. Qualitative classroom research further demonstrates that the manner in which instructional interaction is organized can shape opportunities for learner participation. Conversation-analytic research in high-school EFL classrooms showed that questioning, scaffolding, and other teacher elicitation strategies created different opportunities for student talk, while learner passivity and non-response could restrict meaningful classroom interaction (Farrelly and Sinwongsuwat, 2021). Such evidence is particularly valuable for the present study because it illustrates that classroom dynamics involve situated interactional processes that cannot be reduced to a single numerical cognitive-state variable. Peer interaction and feedback constitute another important component of English pedagogy. A meta-analysis of second-language writing research found that peer feedback can facilitate L2 writing development, although its effectiveness varies according to implementation conditions and study characteristics (Vuogan and Li, 2023). These findings reinforce the importance of instructional and social context, but they also highlight a limitation of the datasets used in the present study: not all potentially relevant classroom variables, such as teacher support, peer relations, motivation, or language anxiety, are directly observed. Consequently, the contextual representation in our framework is restricted to task, prompt, session, and educational variables actually provided by each corpus, while broader classroom and socio-emotional factors are treated as potential confounders and directions for future extension.

### 2.3 Learner-corpus-based language assessment and computational modeling

Learner corpora provide an empirical bridge between second-language research, language assessment, and computational modeling because they preserve authentic learner productions together with proficiency, task, error, or background information. Recent work on learner-corpus pedagogy highlights applications spanning language testing, automatic assessment, materials development, classroom practice, and data-driven learning (Götz and Granger, 2024). This perspective is directly relevant to the present study because the empirical evidence in CSEE, ELLIPSE, SEEFLEX, and LOCSE consists primarily of authentic written or spoken English rather than experimentally measured neural responses. The methodological quality of learner-corpus research depends strongly on corpus design, metadata availability, and appropriate treatment of learner heterogeneity. Recent methodological work has emphasized the need for richer learner metadata, multifactorial study designs, robust quantitative analysis, and transparent corpus construction when learner corpora are used to address questions in second-language acquisition (Paquot, 2024). These considerations motivate our dataset-specific treatment of learner identifiers, repeated observations, task descriptors, demographics, and temporal structure rather than imposing a single observational interpretation on all four corpora. Automated assessment research has increasingly examined how computational systems can support the evaluation of L2 writing. A research synthesis of automated writing evaluation in second-language classrooms found that such systems can influence writing practice and feedback processes, but that learner engagement with automated evaluation depends substantially on individual and contextual factors (Karatay, 2024). A meta-analysis of automated writing evaluation further reported positive overall effects on writing quality while identifying learner population, educational level, and writing genre as important sources of variation (Zhai and Ma, 2023). These findings reinforce the need to model heterogeneous language evidence and contextual information rather than assuming that prediction behavior is invariant across learner groups and tasks. Speaking assessment presents related but distinct challenges. Contemporary accounts treat L2 speaking proficiency as a multidimensional construct involving linguistic and strategic competencies, while recognizing that task, rater, interlocutor, and interactional conditions may introduce construct-irrelevant variation into observed scores (de Jong, 2023). Computational studies of L2 speech have also demonstrated that automated models can approximate human judgments of comprehensibility and generalize across speakers and speaking conditions when the relevant linguistic evidence is appropriately represented (Saito et al., 2023). Together, the writing and speaking literatures justify evaluating the proposed framework across both modalities while retaining externally supplied proficiency or performance annotations as empirical targets. Despite these advances, several methodological requirements remain only partially integrated across existing language-assessment research. Learner-language models must accommodate heterogeneous task and learner conditions, distinguish individual observations from repeated longitudinal measurements, represent contextual associations without interpreting them causally, and communicate uncertainty when predictions are based on limited or noisy evidence. The present study addresses this methodological intersection by combining hierarchical representation learning, sparse low-rank interaction modeling, and separate epistemic and aleatoric uncertainty estimation across written, spoken, cross-sectional, and longitudinal English-learning corpora. This formulation extends computational language assessment while maintaining an explicit distinction between observable educational performance, latent predictive representations, and direct evidence of neuroplastic change.

## 3 Method

### 3.1 Overview

This section presents the methodology developed to characterize dynamic learning patterns and their relationships with instructional conditions in high school English education. The proposed framework, termed HierarchicalFactorizedUncertainty, is motivated by theoretical perspectives on neuroplasticity, which emphasize that learning-related cognitive and behavioral patterns may change over time and vary substantially across individuals. Importantly, the present study does not treat neuroplasticity as a directly observed neural quantity. Instead, neuroplasticity serves as a theoretical lens for modeling adaptive learning dynamics from the educational, behavioral, linguistic, and related cognitive indicators available in the datasets. Accordingly, the framework is intended to identify structured associations and predictive patterns in classroom learning rather than to establish neural mechanisms or causal effects of instruction on brain reorganization.

In section 3.2, we establish the problem formulation and mathematical notation used throughout the study. The formulation distinguishes observable educational variables from latent learning-state representations inferred by the model. These representations summarize learning-related information such as linguistic performance, classroom engagement, instructional context, and other cognitive or behavioral indicators available in the corresponding datasets. They should therefore be interpreted as computational representations of learning states rather than as direct measurements of neural activity or neuroplastic change. The subsection further defines how individual-level observations, classroom-level information, and instructional variables are organized to support the subsequent hierarchical and factorized modeling procedures. The HierarchicalFactorizedUncertainty model is introduced in Section 3.3. It consists of three primary components. The Hierarchical Composition Mapper represents learning information across multiple levels so that individual and broader classroom patterns can be modeled within a unified structure. The Factorized Relation Projector captures compact interactions among learning-related and instructional representations while reducing redundant dependencies. The Uncertainty Propagation Resolver quantifies uncertainty associated with the modeled learning process, with explicit consideration of uncertainty arising from model knowledge and from variability or noise in the observed data. Together, these components provide a structured probabilistic representation of heterogeneous classroom learning dynamics without assuming that the resulting latent representations constitute direct evidence of neuroplastic mechanisms. In 3.4, modular structure learning and sparse representation learning are incorporated to improve the interpretability and computational efficiency of the proposed framework. Modular structure learning organizes complex learning patterns into analyzable substructures, whereas sparse representation learning emphasizes the most informative relations while suppressing redundant interactions. These strategies support the analysis of heterogeneous instructional and learning patterns across classroom settings and facilitate the interpretation of model outputs in the context of high school English education. The proposed methodology combines hierarchical representation learning, factorized interaction modeling, and uncertainty-aware inference to study associations between observed learning indicators and instructional conditions. Its purpose is to provide a neuroplasticity-informed computational account of adaptive classroom learning rather than a direct neural or causal model of neuroplastic change. The following subsections formalize the learning-state variables, model components, uncertainty decomposition, and optimization procedures in detail. For clarity, Table 1 summarizes the principal mathematical symbols used throughout the formulation of the proposed framework.

**Table 1:** Summary of the main mathematical symbols used in the proposed framework.

Symbol	Description
*i*	Index of the basic analysis unit, representing a learner when a stable learner identifier is available, or an individual learner-language record otherwise.
*t*	Dataset-specific observation context, such as a prompt, task, session, or measurement occasion.
$x_{i}^{\left(t\right)}\in {\mathbb{R}}^{D}$	Observed learner-language or performance-related input representation for analysis unit *i* under context *t*.
$I_{i}^{\left(t\right)}\in {\mathbb{R}}^{k}$	Recorded task or contextual feature vector associated with observation (*i, t*).
$h_{i}^{\left(t\right)}\in {\mathbb{R}}^{d}$	Observation-level latent learning-state representation inferred from the observed input.
*L*_*t*_	Aggregate contextual representation summarizing predictive structure shared within context *t*.
$y_{i}^{\left(t\right)}$	Dataset-specific observed prediction target for analysis unit *i* under context *t*.
${ŷ}_{i}^{\left(t\right)}$	Predicted value of the dataset-specific target.
*X*_*t*_	Matrix containing the observed feature vectors associated with context *t*.
$H_{l}^{\left(t\right)}$	Hierarchical representation at level *l* for context *t*.
$r_{i}^{\left(t\right)}$	Low-dimensional factorized interaction representation between the latent learning representation and contextual information.
$a_{i}^{\left(t\right)}$	Integrated representation supplied to the uncertainty-aware predictive component.
Ω	Set of learnable model parameters.
*L*, *R*, *K*	Number of hierarchical levels, attention heads, and low-rank interaction factors, respectively.
*M*	Number of stochastic model realizations used for Monte Carlo dropout inference.
*U*_alea_, *U*_epis_	Aleatoric and epistemic predictive uncertainty, respectively.
λ	Sparse-regularization coefficient controlling the contribution of the low-rank sparsity penalty.

### 3.2 Preliminaries

This subsection formalizes the observed variables, latent representations, contextual information, and prediction setting used by the proposed HierarchicalFactorizedUncertainty framework. Consistent with the scope defined in Section 3.1, neuroplasticity is used as a theoretical motivation for viewing learning-related behavior as adaptive, heterogeneous, and experience-sensitive rather than as a directly observed neural quantity. The formulation therefore distinguishes observable learner-language and educational evidence from latent computational representations inferred by the model. Let *i* index the basic analysis unit used in a dataset. When stable learner identifiers are available, *i* is associated with an individual learner and may occur across multiple observations. When such identifiers are unavailable, *i* indexes an individual learner-language record. Let *t* denote the dataset-specific observation context associated with that record, such as a prompt, task, session, or measurement occasion when such information is available. For longitudinal or sequential data, *t* follows the corresponding temporal order. For cross-sectional or non-sequential data, *t* serves only as a contextual or organizational index and does not imply temporal progression or a causal transition.

For each analysis unit and observation context, the learner-language or performance-related evidence used as predictive input is represented by Equation 1


$$\begin{array}{l} x_{i}^{\left(t\right)}\in {\mathbb{R}}^{D}, \end{array}$$ (1)

where *D* denotes the dimensionality of the observed feature space. Depending on the dataset, $x_{i}^{\left(t\right)}$ may contain representations derived from written or spoken English production together with other performance-related measurements explicitly included as predictive inputs. The exact variables and modalities used for each dataset are specified in Section 4.1. Variables used only for sample characterization or sensitivity analysis are not assumed to form part of $x_{i}^{\left(t\right)}$ unless explicitly stated. These observed variables constitute educational and linguistic evidence and are not interpreted as direct measurements of neuroplastic change. Recorded task and contextual information associated with the same observation is denoted by Equation 2


$$\begin{array}{l} I_{i}^{\left(t\right)}\in {\mathbb{R}}^{k}, \end{array}$$ (2)

where *k* is the dimensionality of the available contextual feature space. Depending on the corpus, $I_{i}^{\left(t\right)}$ may include prompt identifiers, task descriptors, session information, measurement occasions, or other explicitly recorded educational-context variables. Because the datasets used in this study are observational, these variables are treated as conditioning information rather than experimentally manipulated interventions. Accordingly, relationships involving $I_{i}^{\left(t\right)}$ are interpreted as statistical associations and predictive dependencies rather than causal effects of instructional manipulation.

To obtain a compact representation of the observed learner-language evidence, the input vector is mapped to a latent learning-state representation (Equation 3):


$$\begin{array}{l} h_{i}^{\left(t\right)}=\phi_{\Omega }\left(x_{i}^{\left(t\right)}\right),\;h_{i}^{\left(t\right)}\in {\mathbb{R}}^{d}, \end{array}$$ (3)

where ϕ_**Ω**_(·) denotes a learnable representation function parameterized by **Ω**, and *d* is the dimensionality of the latent space. The representation $h_{i}^{\left(t\right)}$ summarizes predictive information contained in the observed educational evidence. It is therefore interpreted as a computational learning-state representation rather than as a psychometrically validated cognitive construct or a direct measurement of neural activity. Its utility is evaluated through downstream prediction and component-level analyses. To represent structure shared across observations associated with the same dataset-specific context, the individual latent representations are aggregated as Equation 4


$$\begin{array}{l} L_{t}=g_{\Omega }\left({\left\{h_{i}^{\left(t\right)}\right\}}_{i=1}^{N_{t}}\right),\;L_{t}\in {\mathbb{R}}^{m}, \end{array}$$ (4)

where *N*_*t*_ denotes the number of observations associated with context index *t*, *m* is the dimensionality of the aggregate contextual representation, and *g*_**Ω**_(·) is a learnable aggregation function. The semantic meaning of *t* and the corresponding grouping of observations are dataset-specific and are described in Section 4.1. Importantly, **L**_*t*_ summarizes shared predictive structure among the observations included in the aggregation and should not be interpreted as an objectively measured classroom state, cognitive state, or neural state. Let $y_{i}^{\left(t\right)}\in Y$ denote the dataset-specific prediction target associated with observation (*i, t*), where the semantic definition and scale of Y depend on the corresponding corpus and prediction task, as specified in Section 4.1. The model estimates the target through Equation 5


$$\begin{array}{l} {ŷ}_{i}^{\left(t\right)}=q_{\Omega }\left(h_{i}^{\left(t\right)},L_{t},I_{i}^{\left(t\right)}\right), \end{array}$$ (5)

where *q*_***Ω***_(·) denotes the predictive mapping implemented by the proposed framework. Model parameters are learned from the training data by minimizing the predictive objective together with the structural and regularization terms introduced in Sections 3.3 and 3.4. The resulting optimization concerns predictive model parameters and does not optimize or estimate causal instructional interventions.

Predictive uncertainty may arise from conceptually distinct sources. We distinguish aleatoric uncertainty from epistemic uncertainty. Aleatoric uncertainty represents observation-dependent variability that remains conditional on a particular model realization, including measurement noise and heterogeneity in the observed outcomes. Epistemic uncertainty represents uncertainty in the learned predictive model arising from finite training evidence, incomplete coverage of the feature space, or uncertainty in parameter estimation. In principle, epistemic uncertainty may be reduced as relevant training evidence increases, whereas aleatoric uncertainty reflects variability associated with the observation process itself.

Using the law of total variance, the predictive uncertainty can be conceptually decomposed as Equation 6


$$\begin{array}{l} \mathrm{Var}\left[Y|x,I,D\right]=\underset{\text{aleatoric uncertainty}}{\underset{︸}{E_{\Omega }\left[\mathrm{Var}\left(Y|x,I,\Omega \right)\right]}}+\underset{\text{epistemic uncertainty}}{\underset{︸}{{\mathrm{Var}}_{\Omega }\left[E\left(Y|x,I,\Omega \right)\right]}}, \end{array}$$ (6)

where D denotes the training data and ***Ω*** denotes a plausible model realization conditioned on the available training evidence. The first term captures the expected predictive variance conditional on a model realization, whereas the second captures variation in the predictive mean across plausible model realizations. Section 3.3 describes how these two uncertainty components are operationalized within the proposed architecture.

On this basis, HierarchicalFactorizedUncertainty models structured predictive dependencies among observed learner-language evidence, latent learning-state representations, aggregate contextual representations, and recorded task or contextual variables. The resulting representations characterize heterogeneous patterns in observable English-learning outcomes. They are not interpreted as direct evidence of neural reorganization or neuroplastic change, and associations involving contextual variables are not interpreted as causal effects of instructional interventions.

### 3.3 HierarchicalFactorizedUncertainty

We introduce HierarchicalFactorizedUncertainty, a computational framework for modeling heterogeneous and uncertain English-learning outcomes in secondary-school educational settings. Consistent with the formulation in Section 3.2, the framework operates on observed learner-language evidence and recorded contextual information and learns latent computational representations for dataset-specific prediction. It does not treat these representations as direct measurements of neural states or neuroplastic change. The framework consists of three jointly optimized components: a Hierarchical Composition Mapper for multi-level representation learning, a Factorized Relation Projector for compact modeling of associations between learned representations and recorded task or contextual variables, and an Uncertainty Propagation Resolver for separately representing aleatoric and epistemic predictive uncertainty. An overview of the proposed HierarchicalFactorizedUncertainty framework is shown in Figure 1.

**Figure 1:**
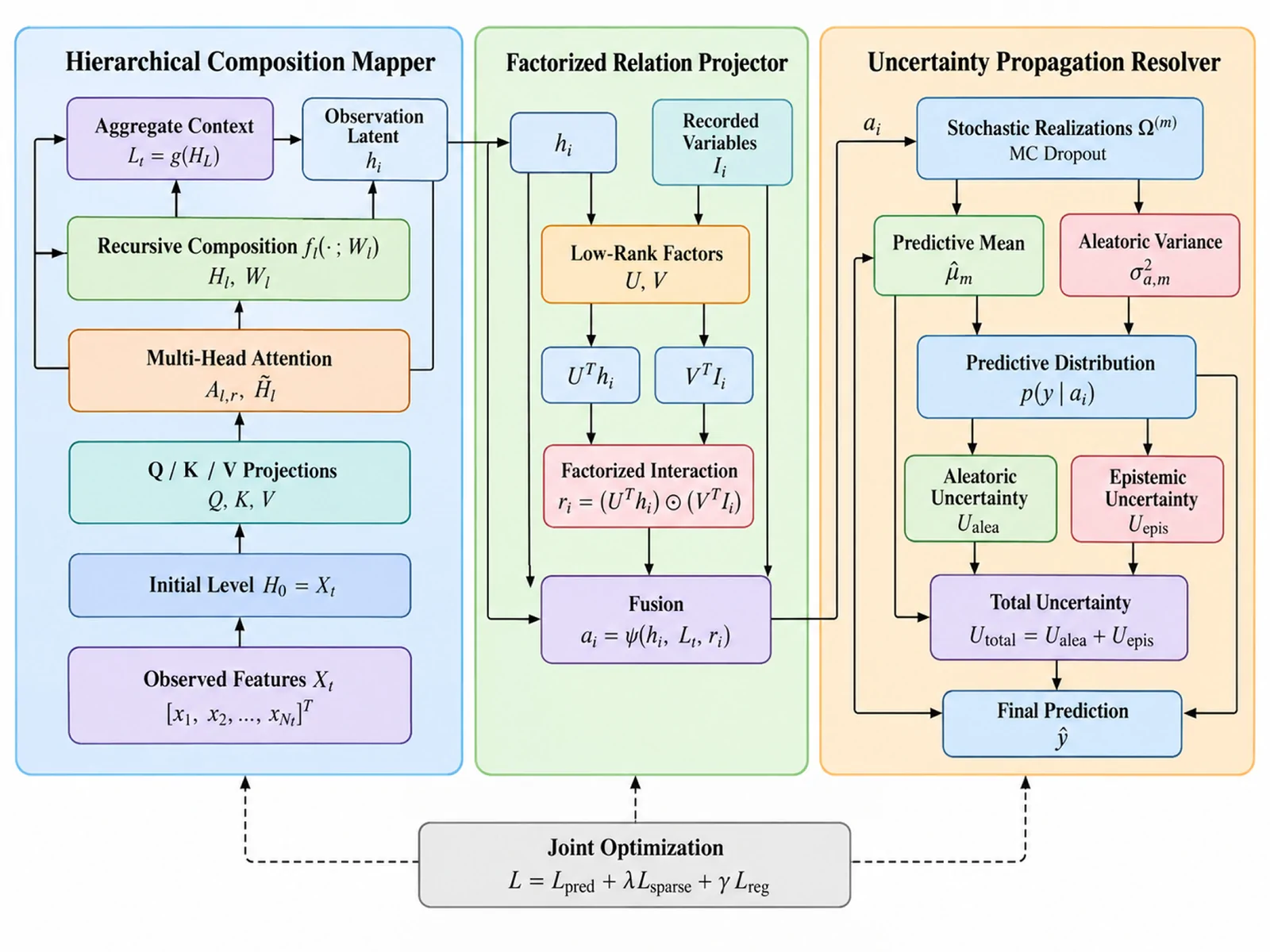
Overview of the proposed HierarchicalFactorizedUncertainty framework. The Hierarchical Composition Mapper transforms the observed feature matrix **X**_*t*_ into observation-level representations $h_{i}^{\left(t\right)}$ and an aggregate contextual representation **L**_*t*_. The Factorized Relation Projector combines $h_{i}^{\left(t\right)}$ with the recorded contextual variables $I_{i}^{\left(t\right)}$ through low-rank factors **U** and **V**, producing the factorized interaction representation $r_{i}^{\left(t\right)}$ and the fused representation $a_{i}^{\left(t\right)}$. The Uncertainty Propagation Resolver then uses stochastic model realizations to obtain the predictive distribution and separately characterize aleatoric and epistemic uncertainty. All components are optimized jointly under the unified predictive objective.

#### 3.3.1 Hierarchical composition mapper

For a dataset-specific context index *t*, let Equation 7


$$\begin{array}{l} X_{t}={\left[x_{1}^{\left(t\right)},x_{2}^{\left(t\right)},\ldots ,x_{N_{t}}^{\left(t\right)}\right]}^{⊤}\in {\mathbb{R}}^{N_{t}\times D} \end{array}$$ (7)

denote the matrix of observed learner-language or performance-related features associated with that context, where *N*_*t*_ is the number of observations included in the corresponding group and *D* is the observed feature dimensionality. The semantic meaning of *t* and the grouping procedure are dataset-specific, as defined in Sections 3.2 and 4.1. The grouping is determined from observed contextual information and does not use target values.

The hierarchical representation is initialized as Equation 8


$$\begin{array}{l} H_{0}^{\left(t\right)}=X_{t}. \end{array}$$ (8)

Successive representation levels are then constructed recursively (Equation 9):


$$\begin{array}{l} H_{l}^{\left(t\right)}=f_{l}\left(H_{l-1}^{\left(t\right)},{\tilde{H}}_{l}^{\left(t\right)};W_{l}\right),\;l=1,\ldots ,L, \end{array}$$ (9)

where **W**_*l*_ denotes the learnable parameters at level *l*, *f*_*l*_(·) is a nonlinear transformation, and ${\tilde{H}}_{l}^{\left(t\right)}$ is an attention-refined representation derived from the preceding level.

For attention head *r*∈{1, …, *R*}, the query, key, and value matrices are defined as


$$\begin{array}{l} Q_{l,r}=H_{l-1}^{\left(t\right)}W_{l,r}^{Q},\;K_{l,r}=H_{l-1}^{\left(t\right)}W_{l,r}^{K},\;V_{l,r}=H_{l-1}^{\left(t\right)}W_{l,r}^{V}. \end{array}$$ (10)

The corresponding attention matrix is


$$\begin{array}{l} A_{l,r}=\mathrm{softmax}\left(\frac{Q_{l,r}K_{l,r}^{⊤}}{\sqrt{d_{r}}}\right), \end{array}$$ (11)

where *d*_*r*_ denotes the dimensionality of the query and key vectors for head *r*. The output of the multi-head attention operation is


$$\begin{array}{l} {\tilde{H}}_{l}^{\left(t\right)}=\mathrm{Concat}\left(A_{l,1}V_{l,1},\ldots ,A_{l,R}V_{l,R}\right)W_{l}^{O}. \end{array}$$ (12)

The final hierarchical representation $H_{L}^{\left(t\right)}$ contains the observation-level latent representations, such that its *i*-th row corresponds to $h_{i}^{\left(t\right)}$ defined in Section 3.2. An aggregate contextual representation is subsequently obtained through


$$\begin{array}{l} L_{t}=g_{\Omega }\left(H_{L}^{\left(t\right)}\right). \end{array}$$ (13)

Through the attention-based transformations, each representation may incorporate information shared among observations associated with the same dataset-specific context. The successive levels therefore represent progressively transformed computational representations rather than biological, neural, or objectively defined classroom hierarchies. The aggregate representation **L**_*t*_ summarizes predictive structure within the corresponding context group and is interpreted according to the dataset-specific grouping defined in Section 4.1.

#### 3.3.2 Factorized relation projector

The Factorized Relation Projector models associations between the learned representation $h_{i}^{\left(t\right)}$ and the recorded task or contextual variables $I_{i}^{\left(t\right)}$ introduced in Section 3.2. These relations are treated as statistical interactions that may contribute to prediction and are not interpreted as causal effects of instructional intervention.

A full bilinear interaction between the two representations can be written as


$$\begin{array}{l} \eta_{i}^{\left(t\right)}={h_{i}^{\left(t\right)}}^{⊤}AI_{i}^{\left(t\right)}, \end{array}$$ (14)

where **A**∈ℝ^*d*×*k*^ is an interaction matrix. Direct estimation of **A** may introduce substantial redundancy when *d* and *k* are large. We therefore impose the low-rank factorization


$$\begin{array}{l} A=UV^{⊤}, \end{array}$$ (15)

where **U**∈ℝ^*d*×*K*^, **V**∈ℝ^*k*×*K*^, and *K*≪min(*d, k*) is the factorization rank.

The corresponding factorized interaction representation is


$$\begin{array}{l} r_{i}^{\left(t\right)}=\left(U^{⊤}h_{i}^{\left(t\right)}\right)⊙\left(V^{⊤}I_{i}^{\left(t\right)}\right),\;r_{i}^{\left(t\right)}\in {\mathbb{R}}^{K}, \end{array}$$ (16)

where ⊙ denotes element-wise multiplication. The corresponding scalar bilinear interaction can be recovered as


$$\begin{array}{l} \eta_{i}^{\left(t\right)}=1^{⊤}r_{i}^{\left(t\right)}. \end{array}$$ (17)

This formulation represents learning-context associations in a compact latent factor space rather than through an unconstrained dense interaction matrix. Sparse regularization on **U** and **V** is introduced in Section 3.4 to suppress weak or redundant interaction factors and improve representation compactness.

The observation-level representation, aggregate contextual representation, and factorized interaction representation are subsequently integrated as


$$\begin{array}{l} a_{i}^{\left(t\right)}=\psi_{\Omega }\left(h_{i}^{\left(t\right)},L_{t},r_{i}^{\left(t\right)}\right), \end{array}$$ (18)

where ψ_***Ω***_(·) denotes a learnable fusion function. The resulting representation $a_{i}^{\left(t\right)}$ is supplied to the uncertainty-aware predictive component.

#### 3.3.3 Uncertainty propagation resolver

Predictive uncertainty can arise from variability associated with the observed outcomes as well as uncertainty in the fitted model. Consistent with the decomposition introduced in Section 3.2, the Uncertainty Propagation Resolver separately represents aleatoric and epistemic uncertainty.

For the score-prediction tasks considered in this study, the predictive head maps the integrated representation $a_{i}^{\left(t\right)}$ to a conditional predictive mean and an observation-dependent variance:


$$\begin{array}{l} {\hat{\mu }}_{i}^{\left(t\right)}=\mu_{\Omega }\left(a_{i}^{\left(t\right)}\right), \end{array}$$ (19)

and


$$\begin{array}{l} {\hat{\sigma }}_{a,i}^{2\left(t\right)}=\mathrm{softplus}\left(s_{\Omega }\left(a_{i}^{\left(t\right)}\right)\right)+\epsilon , \end{array}$$ (20)

where ϵ>0 is a small numerical constant used to maintain strictly positive predictive variance. Conditional on a particular model realization, the predictive distribution is represented as


$$\begin{array}{l} p\left(y_{i}^{\left(t\right)}|a_{i}^{\left(t\right)},\Omega \right)=N\left(y_{i}^{\left(t\right)};{\hat{\mu }}_{i}^{\left(t\right)},{\hat{\sigma }}_{a,i}^{2\left(t\right)}\right). \end{array}$$ (21)

The observation-dependent variance ${\hat{\sigma }}_{a,i}^{2\left(t\right)}$ therefore represents aleatoric uncertainty under the fitted model.

Epistemic uncertainty reflects uncertainty in the learned model arising from finite training evidence or incomplete coverage of the input space. Let


$$\begin{array}{l} \Omega^{\left(m\right)}\sim q\left(\Omega |D\right),\;m=1,\ldots ,M, \end{array}$$ (22)

denote *M* stochastic model realizations from an approximate parameter distribution conditioned on the training data D. In the implementation described in Section 4.2, these realizations are obtained using Monte Carlo dropout during inference.

For realization *m*, the complete predictive path produces


$$\begin{array}{l} {\hat{\mu }}_{i,m}^{\left(t\right)}=\mu_{\Omega^{\left(m\right)}}\left(a_{i,m}^{\left(t\right)}\right),\;{\hat{\sigma }}_{a,i,m}^{2\left(t\right)}=\mathrm{softplus}\left(s_{\Omega^{\left(m\right)}}\left(a_{i,m}^{\left(t\right)}\right)\right)+\epsilon , \end{array}$$ (23)

where $a_{i,m}^{\left(t\right)}$ denotes the integrated representation obtained under the *m*-th stochastic model realization. The predictive distribution marginalized over model uncertainty is approximated by


$$\begin{array}{l} p\left(y_{i}^{\left(t\right)}|X_{t},I_{i}^{\left(t\right)},D\right)\approx \frac{1}{M}\overset{M}{\underset{m=1}{\sum }}N\left(y_{i}^{\left(t\right)};{\hat{\mu }}_{i,m}^{\left(t\right)},{\hat{\sigma }}_{a,i,m}^{2\left(t\right)}\right). \end{array}$$ (24)

Following the law of total variance, aleatoric uncertainty is estimated as the mean conditional predictive variance across stochastic model realizations:


$$\begin{array}{l} U_{\text{alea}}=\frac{1}{M}\overset{M}{\underset{m=1}{\sum }}{\hat{\sigma }}_{a,i,m}^{2\left(t\right)}, \end{array}$$ (25)

whereas epistemic uncertainty is estimated from disagreement among the corresponding predictive means:


$$\begin{array}{l} U_{\text{epis}}={\mathrm{Var}}_{m}\left({\hat{\mu }}_{i,m}^{\left(t\right)}\right). \end{array}$$ (26)

The total predictive uncertainty is consequently represented as


$$\begin{array}{l} U_{\text{total}}=U_{\text{alea}}+U_{\text{epis}}. \end{array}$$ (27)

The final point prediction is obtained from the mean predictive score across the *M* stochastic realizations. In this formulation, aleatoric uncertainty captures observation-dependent variability conditional on the model, whereas epistemic uncertainty captures variability in the predictive mean across plausible model realizations. The stochastic inference procedure and the number of repeated forward passes are specified in Section 4.2.

#### 3.3.4 Unified optimization

The three components are trained jointly rather than through independent module-specific objectives. Let $D_{\text{tr}}$ denote the training set. The predictive objective is defined using the negative log-likelihood


$$\begin{array}{l} L_{\text{pred}}=-\frac{1}{\left|D_{\text{tr}}\right|}\underset{\left(i,t\right)\in D_{\text{tr}}}{\sum }\log p\left(y_{i}^{\left(t\right)}|X_{t},I_{i}^{\left(t\right)}\right). \end{array}$$ (28)

The complete optimization objective is


$$\begin{array}{l} L=L_{\text{pred}}+\lambda L_{\text{sparse}}+\gamma L_{\text{reg}}, \end{array}$$ (29)

where $L_{\text{sparse}}$ denotes the sparse low-rank regularization term introduced in Section 3.4, λ controls its contribution, and $L_{\text{reg}}$ denotes the remaining parameter regularization with coefficient γ. The hierarchical representations, factorized interaction parameters, and uncertainty-aware predictive components are therefore optimized jointly under the same predictive objective. The explicit form of the score-prediction likelihood and the procedures used to select λ and the remaining training hyperparameters are reported in Section 4.2.

### 3.4 Modular architecture and sparse low-rank representation learning

This subsection describes the modular organization of the components introduced in Section 3.3 and the sparse low-rank regularization applied to the factorized interaction structure. The Hierarchical Composition Mapper, Factorized Relation Projector, and Uncertainty Propagation Resolver are treated as functionally distinct but jointly optimized components of a single predictive framework. This organization separates multi-level representation learning, learning-context interaction modeling, and uncertainty-aware prediction, thereby providing explicit computational interfaces for component-level analysis while retaining end-to-end optimization. The modular organization and sparse low-rank learning strategy are summarized in Figure 2.

**Figure 2:**
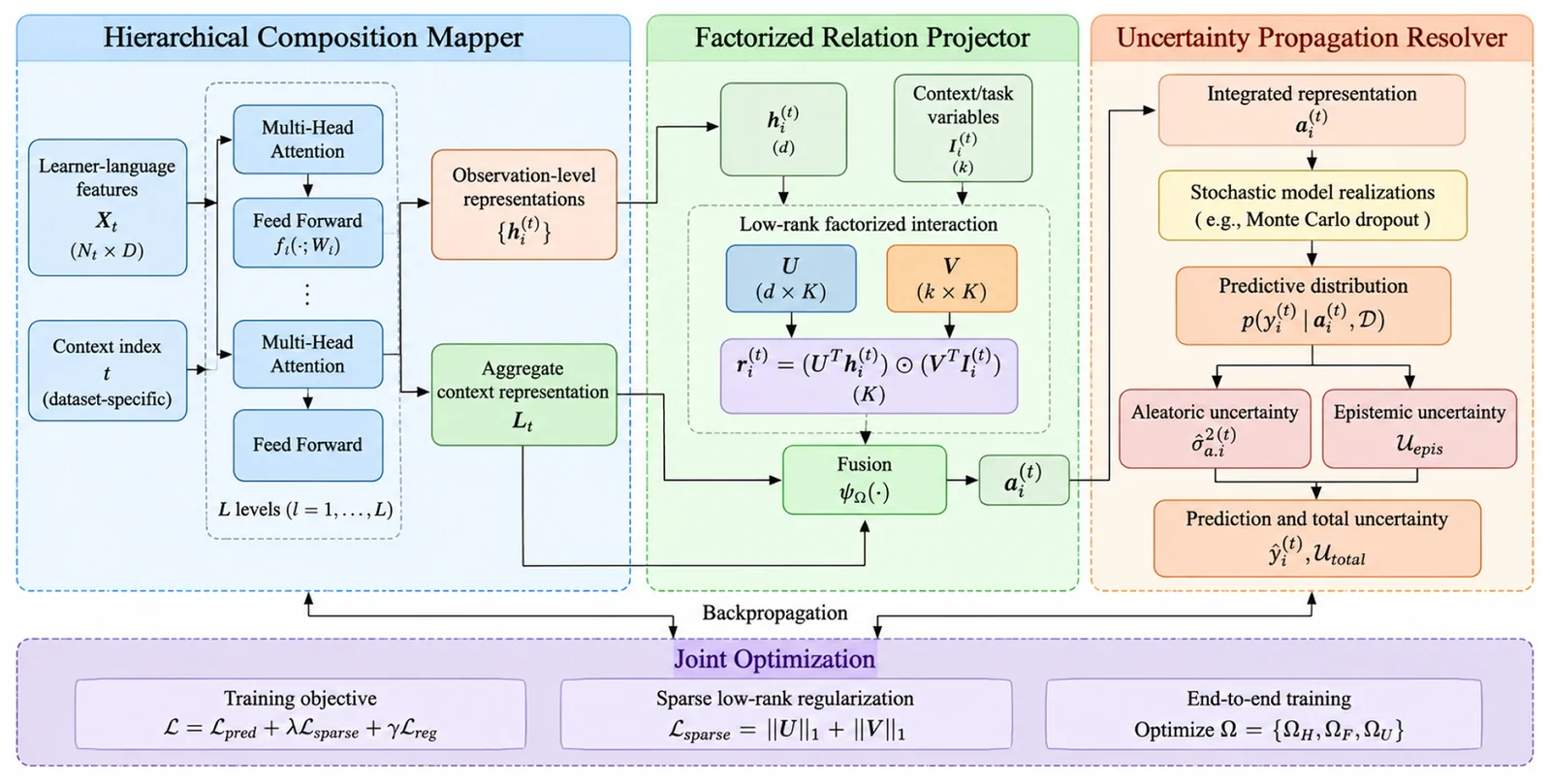
Modular architecture and sparse low-rank representation learning strategy. The Hierarchical Composition Mapper, Factorized Relation Projector, and Uncertainty Propagation Resolver provide functionally distinct interfaces for representation learning, context-interaction modeling, and uncertainty-aware prediction, respectively. Within the factorized component, the interaction matrix is represented as **A** = **UV**^⊤^ with *K*≪min(*d, k*), reducing the interaction parameterization from *dk* to approximately *K*(*d*+*k*). Sparse regularization, $L_{\text{sparse}}=||U|{|}_{1}+||V|{|}_{1}$, encourages selective use of the low-rank factors. Prediction loss and regularization terms are combined in a unified objective, and gradients are propagated through the complete architecture for end-to-end joint optimization.

### 3.5 Modular architecture

Let **Ω**_*H*_, **Ω**_*F*_, and **Ω**_*U*_ denote the parameters associated with the hierarchical, factorized, and uncertainty-aware components, respectively. The complete model parameter set is written as


$$\begin{array}{l} \Omega =\left\{\Omega_{H},\Omega_{F},\Omega_{U}\right\}. \end{array}$$ (30)

The parameters of the fusion function are included in ***Ω***_*F*_ for notational simplicity.

For a dataset-specific context index *t*, the hierarchical component maps the observed feature matrix **X**_*t*_ to observation-level latent representations and an aggregate contextual representation:


$$\begin{array}{l} \left({\left\{h_{i}^{\left(t\right)}\right\}}_{i=1}^{N_{t}},L_{t}\right)=M_{H}\left(X_{t};\Omega_{H}\right), \end{array}$$ (31)

where $M_{H}\left(\cdot \right)$ denotes the Hierarchical Composition Mapper defined in Section 3.3.

The factorized component then combines each observation-level latent representation with the corresponding recorded task or contextual variables:


$$\begin{array}{l} r_{i}^{\left(t\right)}=M_{F}\left(h_{i}^{\left(t\right)},I_{i}^{\left(t\right)};\Omega_{F}\right), \end{array}$$ (32)

where $r_{i}^{\left(t\right)}$ denotes the low-dimensional factorized interaction representation. The observation-level representation, aggregate contextual representation, and factorized interaction representation are subsequently fused as


$$\begin{array}{l} a_{i}^{\left(t\right)}=\psi_{\Omega_{F}}\left(h_{i}^{\left(t\right)},L_{t},r_{i}^{\left(t\right)}\right). \end{array}$$ (33)

The uncertainty-aware component maps the integrated representation to a predictive distribution:


$$\begin{array}{l} p\left(y_{i}^{\left(t\right)}|X_{t},I_{i}^{\left(t\right)},D\right)=M_{U}\left(a_{i}^{\left(t\right)};\Omega_{U},D\right), \end{array}$$ (34)

where $M_{U}\left(\cdot \right)$ implements the aleatoric and epistemic uncertainty mechanisms defined in Section 3.3. In particular, the component produces an observation-dependent predictive variance for aleatoric uncertainty and uses stochastic model realizations at inference time to characterize epistemic uncertainty.

This modular formulation provides explicit interfaces between the three computational functions while allowing all trainable parameters to be optimized jointly. In the present implementation, the number of hierarchical levels *L* is specified as an architectural hyperparameter and remains fixed across datasets and context groups. The modular architecture therefore refers to the functional decomposition of the predictive model rather than to automatic discovery of a variable-depth neural, educational, or cognitive hierarchy. The limitations associated with the fixed hierarchical depth and possible adaptive extensions are discussed in Section 5.

#### 3.5.1 Sparse low-rank representation learning

The Factorized Relation Projector introduced in Section 3.3 represents the interaction matrix between the latent learning-state representation and the recorded contextual feature vector as


$$\begin{array}{l} A=UV^{⊤}, \end{array}$$ (35)

where **U**∈ℝ^*d*×*K*^ and **V**∈ℝ^*k*×*K*^ project the two input spaces into a shared *K*-dimensional factor space. When *K*≪min(*d, k*), the low-rank factorization reduces the number of interaction parameters from *dk* to approximately *K*(*d*+*k*).

Low rank reduces parameter redundancy but does not by itself encourage selective use of the factor dimensions. We therefore impose an ℓ_1_ penalty on the factor matrices:


$$\begin{array}{l} L_{\text{sparse}}=||U|{|}_{1}+||V|{|}_{1}. \end{array}$$ (36)

This term is incorporated into the unified training objective defined in Section 3.3, where λ controls the contribution of sparse low-rank regularization.

The ℓ_1_ penalty encourages weak coefficients in **U** and **V** to shrink toward zero, thereby concentrating the interaction structure on a smaller subset of predictive factors. In combination with the low-rank constraint, this mechanism suppresses redundant dependencies and yields a more compact representation of associations between latent learning representations and recorded task or contextual variables. Because these associations are estimated from observational educational data, nonzero factor coefficients are interpreted as predictive relationships rather than as evidence of causal instructional effects or direct neuroplastic mechanisms.

The regularization coefficient λ determines the trade-off between predictive fit and sparsity. A value that is too small provides limited suppression of weak interactions, whereas an excessively large value may remove informative factors and reduce predictive performance. We therefore select λ using the validation procedure described in Section 4.2. The candidate search range, validation criterion, and dataset-specific selected values are reported there for reproducibility.

#### 3.5.2 Joint learning strategy

Although the three components have distinct computational roles, they are trained end-to-end rather than optimized independently. For each training example within a dataset-specific context group, the Hierarchical Composition Mapper constructs observation-level and aggregate contextual representations, the Factorized Relation Projector models compact learning-context associations, and the Uncertainty Propagation Resolver produces an uncertainty-aware predictive distribution.

Gradients from $L_{\text{pred}}$ are propagated through the complete computational path, while $L_{\text{sparse}}$ specifically regularizes the low-rank factor matrices **U** and **V**. The remaining parameter regularization term is applied according to the unified objective defined in Section 3.3. This joint strategy retains the functional separation of the three components while allowing them to learn representations that are optimized for the same dataset-specific predictive task.

The resulting architecture should therefore be understood as a structured predictive model of heterogeneous English-learning observations. Its modular components correspond to computational functions representation learning, context-interaction modeling, and uncertainty estimation rather than to separate biological or neuroplastic mechanisms.

## 4 Experimental setup

### 4.1 Datasets and task definition

The empirical evaluation uses four complementary learner corpora: the Chinese Student English Essay (CSEE) dataset, the English Language Learner Insight, Proficiency and Skills Evaluation (ELLIPSE) corpus, the Corpus of Secondary School English as a Foreign Language Exams (SEEFLEX), and the Longitudinal Corpus of Spoken English (LOCSE) (Xiao et al., 2025; Crossley et al., 2023; Pauls, 2026; Abe and Kondo, 2019). Together, these corpora provide authentic written or spoken English production, externally defined performance or proficiency outcomes, recorded task or contextual information, and, in LOCSE, repeated longitudinal observations. They therefore support evaluation of the proposed framework across heterogeneous secondary-school English-learning settings while restricting the empirical targets to observable language-performance outcomes.

CSEE is an English writing corpus collected from Chinese high-school students in authentic examination settings (Xiao et al., 2025). The original study reports 13,372 essays collected in collaboration with 29 high schools and elicited by two final-examination writing prompts. The publicly released version used in the present study contains 13,270 essays together with prompt identifiers, essay texts, and teacher-assigned scores. The scoring rubric follows the Chinese National College Entrance Examination writing criteria and includes an Overall score with a nominal range of 0–20, together with Content, Language, and Structure subscores. The primary CSEE task is prediction of the teacher-assigned Overall writing score from the essay text and recorded prompt information. Because the analytic Content, Language, and Structure scores contribute directly to the holistic assessment, they are excluded from the predictive input when the Overall score is used as the target, thereby preventing direct label leakage. The essay text constitutes the observed learner-language evidence, whereas the prompt identifier is treated as contextual information. The public release does not provide stable individual-level demographic information such as age, gender, or socioeconomic status, and these characteristics are neither reconstructed nor inferred. Personally identifiable student and school information was removed prior to public release. Accordingly, CSEE is treated as a cross-sectional corpus in the present study: each essay constitutes an analysis record, and no learner-level longitudinal ordering is assumed.

ELLIPSE is an open learner corpus containing 6,482 essays written by English language learners in Grades 8–12 during standardized educational assessment in the United States (Crossley et al., 2023). The corpus contains responses to multiple independent writing prompts and provides human-assigned holistic English-proficiency scores together with six analytic dimensions: Cohesion, Syntax, Vocabulary, Phraseology, Grammar, and Conventions. The primary ELLIPSE task is prediction of the Overall English proficiency score from the learner essay and available prompt information. As in CSEE, the analytic trait scores are not included as predictive inputs when the Overall score is the target because their use would introduce direct information from constituent rating dimensions. They are retained only for descriptive or secondary analyses. ELLIPSE additionally provides learner metadata including gender, grade level, economic status, and race/ethnicity. These attributes are used for population characterization and, where appropriate, stratified robustness analyses, but they are not included in the default predictive feature set. Because the corpus includes Grade 8 as well as Grades 9–12, it is treated as a complementary secondary-school benchmark that broadens the grade-level coverage of the empirical evaluation. No repeated temporal learning trajectory is assumed for ELLIPSE.

SEEFLEX comprises authentic curriculum-based EFL examinations collected from upper-secondary students in Germany (Pauls, 2026). The current corpus release contains 575 transcribed examinations comprising 1,979 individual texts and approximately 625,000 words. The corpus represents multiple curriculum-based writing tasks and includes text-, task-, and learner-level metadata. Available learner information includes standardized receptive vocabulary assessment, a cognition measure, reading habits, social-background information, and indicators of language experience and English proficiency. In the present study, the written English productions provide the principal learner-language evidence, while recorded task descriptors provide contextual information. The standardized English-language proficiency information supplied with the corpus is used as the externally defined learner-performance outcome. Cognition, reading habits, language experience, and social-background variables are reserved for sample characterization and sensitivity analyses unless explicitly stated otherwise and are not included in the default predictive feature set. SEEFLEX contains multiple texts from many individual learners. Learner identity is therefore retained for grouped data partitioning so that texts produced by the same learner do not appear in both development and test partitions. Although the corpus provides repeated task observations for individual learners, these records do not constitute a regularly spaced longitudinal panel. Their task or text indices are therefore treated as non-temporal observation indices rather than as ordered learning-state transitions.

LOCSE provides the longitudinal component of the evaluation and follows Japanese senior-high-school learners studying English in an EFL environment (Abe and Kondo, 2019). The complete longitudinal subset used in the present study contains 104 learners who participated at all eight data-collection occasions over a 23-month period. This subset comprises 47 male and 57 female learners, who were 15 or 16 years old at the beginning of data collection. All participants were native speakers of Japanese and studied English in a senior-high-school environment. At each measurement occasion, learners completed a monologic English-speaking assessment consisting of multiple speaking tasks. Their spoken responses were transcribed to form the longitudinal corpus. Speaking proficiency was independently assessed using the Telephone Standard Speaking Test framework, in which certified raters assign holistic proficiency levels based on dimensions such as functional ability, sentence structure, accuracy, and content. The primary LOCSE task is prediction of the externally assigned holistic speaking-proficiency level from the learner's spoken-language representation together with recorded task information and measurement occasion. Unlike the other three corpora, the index *t* has an explicit temporal interpretation in LOCSE. For each learner,


$$\begin{array}{l} t\in \left\{1,\ldots ,8\right\}, \end{array}$$ (37)

corresponding to the eight longitudinal measurement occasions. The complete subset therefore contains 832 learner-occasion observations before task-level expansion. This structure enables evaluation of predictive performance across repeated observations of spoken English within learners over time. The resulting longitudinal variation is interpreted as observable language-development evidence rather than as a direct measurement of neural change.

Table 2 summarizes the empirical scope, observational structure, and prediction target of the four datasets.

**Table 2:** Characteristics and task definitions of the four datasets used in the empirical evaluation.

Dataset	Analysis sample	Longitudinal points	Observed English-learning evidence	Primary prediction target
CSEE	13,270 public-release essays; 2 prompts	–	Essay text and prompt information	Teacher-assigned Overall writing score
ELLIPSE	6,482 essays	–	Essay text and prompt information	Overall English proficiency score
SEEFLEX	575 examinations; 1,979 texts	–	Curriculum-based writing and task/context metadata	Externally provided English-proficiency measure
LOCSE	104 learners; 832 learner-occasion observations	8 over 23 months	Transcribed spoken English, speaking task, and measurement occasion	Holistic speaking-proficiency level

A dash in the longitudinal-points column indicates that no regularly repeated temporal trajectory is assumed.

Across the four corpora, the notation introduced in Section 3.2 is instantiated only from variables explicitly available in the corresponding data source. The observed vector $x_{i}^{\left(t\right)}$ contains the learner-language or performance-related evidence used as predictive input, whereas $I_{i}^{\left(t\right)}$ contains recorded prompt, task, session, measurement-occasion, or other available contextual information. Demographic, social-background, and auxiliary cognitive variables are not included in the default predictive feature set unless explicitly stated otherwise. The target $y_{i}^{\left(t\right)}$ denotes the externally supplied performance or proficiency outcome defined for the corresponding corpus. The interpretation of the indices is adapted to the observational structure of each dataset. For CSEE and ELLIPSE, the analysis unit is an individual essay record and no temporal ordering is imposed. For SEEFLEX, repeated texts may be associated with the same learner, but the corresponding observation indices are non-temporal and learner identity is used primarily to preserve grouping during data partitioning. For LOCSE, *i* identifies a learner and *t* identifies one of the eight longitudinal measurement occasions. For the hierarchical component, the context index used to construct **X**_*t*_ and the aggregate contextual representation **L**_*t*_ is determined only from recorded dataset-specific contextual information and not from the prediction target. For the non-longitudinal corpora, this index has no temporal interpretation; for LOCSE, it follows the longitudinal measurement occasion. The exact construction of context-grouped inputs within the development and test partitions is described in Section 4.2. All four datasets are used exclusively for secondary analysis. The present study involves no new student recruitment, educational intervention, neural or physiological measurement, or collection of personally identifiable information. Only de-identified or access-controlled research data made available by the original data providers are used, and no attempt is made to re-identify individual learners. Original consent procedures, anonymization practices, access conditions, and available ethics information are reported in the Ethics Statement and Data Availability Statement. Where a source does not publicly report a specific institutional-review identifier, no approval identifier is inferred.

### 4.2 Experimental details

All experiments were implemented in Python 3.10.13 using PyTorch 2.4.1 and CUDA 12.1 with cuDNN 9.1. Training was performed on a workstation equipped with a single NVIDIA A100 GPU with 80 GB of GPU memory and 128 GB of system RAM under Ubuntu 22.04 LTS. Automatic mixed-precision training was enabled through the PyTorch AMP framework. Unless otherwise specified, all compared methods were evaluated using the same dataset partitions and repeated-run protocol.

#### 4.2.1 Text representation and preprocessing

The four corpora contain written essays or transcribed spoken English. Original spelling, punctuation, grammatical errors, and lexical choices were preserved because these characteristics constitute meaningful evidence of learner proficiency. We did not apply automatic grammar correction, spelling correction, synonym replacement, back-translation, or other text-augmentation procedures that could alter the observed proficiency signal. Raw English texts were converted into fixed-dimensional representations using a pretrained RoBERTa-base language encoder. Texts were tokenized using the corresponding subword tokenizer. Sequences exceeding the maximum input length were divided into windows of at most 512 tokens with an overlap of 64 tokens. A representation was extracted for each window, and the window-level representations were mean-pooled to obtain a 768-dimensional text vector for each essay or spoken transcript. For the proposed HierarchicalFactorizedUncertainty framework, the pretrained RoBERTa-base encoder was kept fixed and the downstream hierarchical, factorized, fusion, and uncertainty-aware components were trained on top of these representations. Prompt, task, session, and measurement-occasion information, when available, was represented separately as the contextual vector $I_{i}^{\left(t\right)}$. Numerical contextual variables were standardized using statistics estimated from the corresponding training split only. Categorical contextual variables were mapped to learned embeddings, with an additional category reserved for previously unseen values. Missing numerical values, when present, were imputed using the median estimated from the corresponding training split and accompanied by a missingness indicator. Demographic, social-background, and auxiliary cognitive attributes described in Section 4.1 were not included in the default predictive feature set.

#### 4.2.2 Data partitioning and validation protocol

For each dataset, approximately 20% of the corpus-specific partitioning units were reserved as a held-out test set, and the remaining 80% formed the development set. The test set was not used for model selection, early stopping, or hyperparameter tuning. The outer development-test split was generated using a fixed partition seed of 2026. The partitioning unit was adapted to the observational structure of each corpus. For CSEE and ELLIPSE, which are treated as cross-sectional essay corpora, splitting was performed at the essay-record level while approximately preserving the distributions of writing prompts and target scores. Because stable learner identifiers are not available in the public CSEE release, no unreported learner-level grouping was assumed. For SEEFLEX, all texts produced by the same learner were assigned to the same partition. For LOCSE, partitioning was performed at the learner level, and all eight longitudinal observations from a given learner were retained in a single partition. These learner-grouped procedures prevent repeated observations from the same SEEFLEX or LOCSE learner from appearing in both development and test data. Hyperparameter selection was conducted exclusively within the 80% development set using five-fold cross-validation. The same dataset-specific grouping constraints were retained within each fold. For score-based outcomes, approximate target-distribution balance was obtained using score strata derived from the corresponding training data. Four folds were used for parameter estimation and the remaining fold for validation, with each fold serving once as the validation fold. Hyperparameters were selected according to the lowest mean validation predictive negative log-likelihood across the five folds. When two configurations produced comparable likelihood values, the configuration with the better validation agreement or error metric was selected. The final model was subsequently retrained on the complete development set, with the training duration determined from the median best-performing epoch identified during cross-validation.

#### 4.2.3 Prediction formulation and training objective

The externally supplied writing- or speaking-performance outcome $y_{i}^{\left(t\right)}$ was retained on its native numerical or ordered scale rather than being converted into arbitrary proficiency categories. For optimization, target scores were standardized using statistics estimated from the corresponding training split and were transformed back to their original scale for evaluation. The predictive head produced a conditional predictive mean ${\hat{\mu }}_{i}^{\left(t\right)}$ and an observation-dependent variance ${\hat{\sigma }}_{a,i}^{2\left(t\right)}$. Consistent with the aleatoric uncertainty formulation in Section 3.3, the predictive loss was implemented as a heteroscedastic Gaussian negative log-likelihood:


$$\begin{array}{l} L_{\text{pred}}=\frac{1}{\left|D_{\text{tr}}\right|}\underset{\left(i,t\right)\in D_{\text{tr}}}{\sum }\left[\frac{{\left(y_{i}^{\left(t\right)}-{\hat{\mu }}_{i}^{\left(t\right)}\right)}^{2}}{2{\hat{\sigma }}_{a,i}^{2\left(t\right)}}+\frac{1}{2}\log{\hat{\sigma }}_{a,i}^{2\left(t\right)}\right]. \end{array}$$ (38)

Training followed the unified objective defined in Section 3.3, with the sparse low-rank regularization term defined in Section 3.4:


$$\begin{array}{l} L=L_{\text{pred}}+\lambda L_{\text{sparse}}+\gamma L_{\text{reg}}. \end{array}$$ (39)

#### 4.2.4 Model configuration and optimization

The 768-dimensional text representation was projected to the latent dimensionality *d* = 128 before entering the Hierarchical Composition Mapper. Unless otherwise specified, the proposed model used *L* = 3 hierarchical levels, four attention heads, and a factorization rank of *K* = 32. Contextual variables were projected to a common representation before entering the Factorized Relation Projector. Model parameters were initialized using Xavier initialization and optimized using Adam with an initial learning rate of 1 × 10^−4^. The regularization coefficient was set to γ = 1 × 10^−5^. The batch size was 64 and the maximum training duration was 100 epochs. An adaptive learning-rate scheduler reduced the learning rate by a factor of 0.5 after five consecutive epochs without improvement in validation negative log-likelihood, with a minimum learning rate of 1 × 10^−6^. Early stopping was applied after 12 consecutive epochs without validation improvement. Dropout with probability *p* = 0.5 was applied to the hierarchical, factorized, fusion, and prediction layers. The dropout probability remained fixed throughout training. The pretrained text encoder was evaluated deterministically and was not included in the stochastic dropout mechanism.

#### 4.2.5 Sparse-regularization tuning

The coefficient λ controlling the sparse low-rank regularization was selected independently for each dataset using the five-fold development-set procedure described above. The candidate grid was


$$\begin{array}{l} \lambda \in \left\{10^{-5},5\times 10^{-5},10^{-4},5\times 10^{-4},10^{-3},5\times 10^{-3},10^{-2}\right\}. \end{array}$$ (40)

The selected values were


$$\begin{array}{l} \lambda_{\text{CSEE}}=5\times 10^{-4},\;\lambda_{\text{ELLIPSE}}=10^{-3}, \end{array}$$

$$\begin{array}{l} \lambda_{\text{SEEFLEX}}=5\times 10^{-4},\;\lambda_{\text{LOCSE}}=10^{-4}. \end{array}$$ (41)

No test-set result was consulted during this selection procedure. Reporting the complete candidate range and dataset-specific selected values makes the sparse-regularization tuning procedure explicit and reproducible.

#### 4.2.6 Uncertainty estimation

Epistemic uncertainty was estimated using Monte Carlo dropout. At inference time, dropout remained active in the trainable HierarchicalFactorizedUncertainty components, and *M* = 30 stochastic forward passes were performed for each test prediction. For stochastic realization *m*, the predictive head produced a conditional predictive mean ${\hat{\mu }}_{i,m}^{\left(t\right)}$ and an observation-dependent variance ${\hat{\sigma }}_{a,i,m}^{2\left(t\right)}$. Consistent with Section 3.3, aleatoric uncertainty was estimated as the mean conditional predictive variance across the stochastic model realizations:


$$\begin{array}{l} U_{\text{alea}}=\frac{1}{M}\overset{M}{\underset{m=1}{\sum }}{\hat{\sigma }}_{a,i,m}^{2\left(t\right)}, \end{array}$$ (42)

whereas epistemic uncertainty was estimated from the variance of the corresponding predictive means:


$$\begin{array}{l} U_{\text{epis}}={\mathrm{Var}}_{m}\left({\hat{\mu }}_{i,m}^{\left(t\right)}\right). \end{array}$$ (43)

The total predictive uncertainty was represented as


$$\begin{array}{l} U_{\text{total}}=U_{\text{alea}}+U_{\text{epis}}. \end{array}$$ (44)

The final point prediction was the mean of the *M* predictive means:


$$\begin{array}{l} {\hat{y}}_{i}^{\left(t\right)}=\frac{1}{M}\overset{M}{\underset{m=1}{\sum }}{\hat{\mu }}_{i,m}^{\left(t\right)}. \end{array}$$ (45)

This procedure operationalizes the aleatoric-epistemic decomposition introduced in Sections 3.2 and 3.3 without mixing observation-level variance with between-realization disagreement.

#### 4.2.7 Evaluation metrics

Because the prediction tasks involve numerical or ordered English-performance outcomes, classification metrics such as Accuracy, Precision, Recall, and F1-score were not used as primary evaluation criteria. Performance was evaluated using mean absolute error (MAE), root mean squared error (RMSE), Spearman's rank correlation coefficient (ρ), and quadratic weighted kappa (QWK) when the original target was defined on a finite ordered rating scale. MAE and RMSE quantify the magnitude of score-prediction errors, with lower values indicating better performance. Spearman's ρ evaluates whether predicted scores preserve the ranking of learner performance. QWK evaluates agreement between predicted and reference ratings while assigning larger penalties to larger ordinal disagreements. For QWK calculation, predictions were clipped to the valid score range of the corresponding dataset and mapped to the nearest admissible rating value. No such rounding was applied when calculating MAE, RMSE, or Spearman's ρ.

#### 4.2.8 Repeated-run analysis and reproducibility

To quantify variability associated with stochastic optimization, each final model configuration was trained independently using 10 predetermined random seeds:


$$\begin{array}{l} \left\{11,23,37,53,71,89,107,131,151,173\right\}. \end{array}$$ (46)

The same seed values were applied to the Python, NumPy, PyTorch, and CUDA random-number generators. Dataset partitions were held fixed across repeated runs so that the resulting standard deviations primarily reflected stochastic training variability rather than changes in test-set composition. All compared methods were evaluated using the same development and test partitions. Results in Tables 2–5 are reported as mean ± standard deviation over these 10 training runs.

#### 4.2.9 Statistical significance testing

Pairwise two-sided Welch independent-samples *t*-tests were conducted between HierarchicalFactorizedUncertainty and each comparison baseline using the run-level metric values obtained from the 10 repeated experiments. Separate comparisons were performed for every dataset and every reported evaluation metric. The null hypothesis for each comparison was that the mean run-level performance of the proposed method and the corresponding baseline was equal. Statistical significance was initially evaluated at α = 0.05. Because multiple baselines were compared with the proposed model for each dataset-metric combination, Holm-Bonferroni correction was applied within each comparison family. Adjusted significance levels are indicated in Tables 2, 3 using ^*^, ^**^, and ^***^ for adjusted *p* < 0.05, *p* < 0.01, and *p* < 0.001, respectively. Exact adjusted *p*-values, 95% confidence intervals, and Hedges' *g* effect sizes are provided in the supplementary statistical results. These tests assess the reproducibility of model-performance differences across stochastic training runs under a fixed test partition. They therefore characterize optimization-related run-to-run variability rather than population-level sampling uncertainty.

**Table 3:** Comparison with strong baselines and pretrained language models on CSEE and ELLIPSE.

Model	CSEE				ELLIPSE			
	MAE ↓	RMSE ↓	*ρ* ↑	QWK ↑	MAE ↓	RMSE ↓	*ρ* ↑	QWK ↑
Ridge Regression	1.46 ± 0.05^***^	1.91 ± 0.06^***^	0.768 ± 0.012^***^	0.774 ± 0.011^***^	0.443 ± 0.012^***^	0.581 ± 0.016^***^	0.637 ± 0.014^***^	0.621 ± 0.013^***^
SVR	1.37 ± 0.04^***^	1.81 ± 0.05^***^	0.789 ± 0.010^***^	0.794 ± 0.009^***^	0.414 ± 0.011^***^	0.548 ± 0.014^***^	0.665 ± 0.012^***^	0.649 ± 0.011^***^
BiLSTM-Attention	1.32 ± 0.05^***^	1.74 ± 0.06^***^	0.801 ± 0.011^***^	0.807 ± 0.010^***^	0.398 ± 0.014^***^	0.530 ± 0.017^***^	0.680 ± 0.014^***^	0.663 ± 0.013^***^
BERT	1.25 ± 0.04^***^	1.66 ± 0.05^***^	0.817 ± 0.009^***^	0.823 ± 0.008^***^	0.369 ± 0.010^***^	0.499 ± 0.013^***^	0.709 ± 0.011^***^	0.701 ± 0.010^***^
RoBERTa	1.20 ± 0.03^***^	1.60 ± 0.04^***^	0.829 ± 0.008^**^	0.834 ± 0.007^**^	0.352 ± 0.009^***^	0.480 ± 0.012^***^	0.726 ± 0.010^***^	0.716 ± 0.009^***^
DeBERTa-v3	1.16 ± 0.04 ^**^	1.56 ± 0.04 ^**^	0.838 ± 0.009	0.841 ± 0.008 ^*^	0.337 ± 0.009 ^**^	0.463 ± 0.011 ^**^	0.741 ± 0.009 ^**^	0.731 ± 0.009 ^**^
**HierarchicalFactorizedUncertainty**	**1.10 ± 0.03**	**1.49 ± 0.04**	**0.847 ± 0.007**	**0.852 ± 0.006**	**0.319 ± 0.008**	**0.445 ± 0.010**	**0.756 ± 0.008**	**0.748 ± 0.008**

MAE and RMSE are lower-is-better (↓), whereas Spearman's ρ and QWK are higher-is-better (↑). Results are reported as mean ± standard deviation over 10 repeated training runs. Asterisks attached to baseline results indicate a statistically significant difference from HierarchicalFactorizedUncertainty after Holm-Bonferroni correction: ^*^p < 0.05, ^**^p < 0.01, and ^***^p < 0.001. Underlined values indicate the second-best performance among all comparison methods. Bolded values indicate the optimal values.

### 4.3 Comparison with strong baselines and pretrained language models

We compare HierarchicalFactorizedUncertainty with representative conventional, recurrent, and pretrained language-model baselines under the unified experimental protocol described in Section 4.2. The comparison includes Ridge Regression (Hoerl and Kennard, 1970) and Support Vector Regression (SVR) (Drucker et al., 1997) as conventional scoring baselines, a bidirectional LSTM with attention (BiLSTM-Attention) (Zhou et al., 2016; Taghipour and Ng, 2016) as a recurrent neural baseline, and BERT (Devlin et al., 2019), RoBERTa (Liu et al., 2019), and DeBERTa-v3 (He et al., 2023) as pretrained Transformer baselines. All baseline results were obtained under the dataset partitions, development-set tuning restrictions, and repeated-run protocol used in the present study rather than being taken from previously published performance values. For Ridge Regression and SVR, the same fixed-dimensional text representations used by the proposed framework were supplied as predictive input. The pretrained Transformer baselines were equipped with task-specific scoring heads, with model selection and hyperparameter tuning performed exclusively on the development partitions. All methods used identical development and test partitions and were evaluated using the same 10-run protocol. This design enables comparison under a common empirical setting despite differences among the model architectures.

Tables 3, 4 report predictive performance across the four corpora. On the two writing corpora in Table 3, HierarchicalFactorizedUncertainty achieves the best mean value for every reported metric. On CSEE, the proposed method obtains an MAE of 1.10 and an RMSE of 1.49, together with a Spearman's ρ of 0.847 and a QWK of 0.852. Relative to DeBERTa-v3, which is the strongest comparison baseline in terms of mean performance, this corresponds to reductions of approximately 5.2% in MAE and 4.5% in RMSE, while QWK increases from 0.841 to 0.852. These differences remain statistically significant after Holm-Bonferroni correction for MAE, RMSE, and QWK. The difference in Spearman's ρ, from 0.838 to 0.847, does not remain statistically significant after correction. Thus, the proposed framework shows a clearer advantage in absolute prediction error and score agreement than in rank ordering on CSEE. A similar mean-performance pattern is observed on ELLIPSE. HierarchicalFactorizedUncertainty achieves an MAE of 0.319, an RMSE of 0.445, a Spearman's ρ of 0.756, and a QWK of 0.748. The pretrained Transformer baselines also outperform the conventional and recurrent baselines across the reported metrics, which is consistent with the usefulness of contextualized language representations for English-performance prediction. The proposed framework provides additional mean improvements over DeBERTa-v3, and the corresponding differences shown in Table 3 remain statistically significant after correction. Because the six analytic ELLIPSE trait scores are excluded from the primary predictive inputs when the Overall score is the target, these gains cannot be attributed to direct reuse of constituent rating labels.

**Table 4:** Comparison with strong baselines and pretrained language models on SEEFLEX and LOCSE.

Model	SEEFLEX			LOCSE			
	MAE ↓	RMSE ↓	*ρ* ↑	MAE ↓	RMSE ↓	*ρ* ↑	QWK ↑
Ridge Regression	7.42 ± 0.31^***^	9.66 ± 0.38^***^	0.602 ± 0.022^***^	0.643 ± 0.032^***^	0.806 ± 0.040^***^	0.612 ± 0.025^***^	0.604 ± 0.024^***^
SVR	6.91 ± 0.28^***^	9.08 ± 0.34^***^	0.635 ± 0.019^***^	0.591 ± 0.029^***^	0.754 ± 0.036^***^	0.649 ± 0.022^***^	0.641 ± 0.021^***^
BiLSTM-Attention	6.74 ± 0.33^***^	8.86 ± 0.41^***^	0.648 ± 0.021^***^	0.568 ± 0.031^***^	0.726 ± 0.039^***^	0.666 ± 0.024^***^	0.659 ± 0.022^***^
BERT	6.22 ± 0.27^***^	8.19 ± 0.34^***^	0.684 ± 0.018^***^	0.523 ± 0.027^***^	0.677 ± 0.034^***^	0.703 ± 0.020^***^	0.695 ± 0.019^***^
RoBERTa	5.97 ± 0.25^**^	7.90 ± 0.31^**^	0.701 ± 0.016^**^	0.497 ± 0.025^**^	0.648 ± 0.031^**^	0.722 ± 0.018^**^	0.716 ± 0.018^**^
DeBERTa-v3	5.78 ± 0.24 ^*^	7.67 ± 0.30 ^*^	0.714 ± 0.017 ^*^	0.482 ± 0.024 ^*^	0.631 ± 0.030 ^*^	0.735 ± 0.017 ^*^	0.728 ± 0.017 ^*^
**HierarchicalFactorizedUncertainty**	**5.41 ± 0.22**	**7.21 ± 0.28**	**0.738 ± 0.015**	**0.451 ± 0.022**	**0.586 ± 0.028**	**0.759 ± 0.016**	**0.751 ± 0.015**

QWK is reported for LOCSE because its target is an ordered speaking-proficiency level; it is not reported for the continuous SEEFLEX proficiency target. Results are reported as mean ± standard deviation over 10 repeated training runs. Asterisks attached to baseline results indicate a statistically significant difference from HierarchicalFactorizedUncertainty after Holm-Bonferroni correction: ^*^p < 0.05, ^**^p < 0.01, and ^***^p < 0.001. Underlined values indicate the second-best performance among all comparison methods. Bolded values indicate the optimal values.

Table 4 evaluates the models on SEEFLEX and LOCSE, which represent different forms of learner and contextual heterogeneity. SEEFLEX contains heterogeneous curriculum-based writing tasks together with repeated texts from individual learners, whereas LOCSE contains repeated longitudinal speaking observations across eight measurement occasions. On SEEFLEX, HierarchicalFactorizedUncertainty achieves the lowest prediction errors and the highest rank correlation. Compared with DeBERTa-v3, MAE decreases from 5.78 to 5.41, RMSE decreases from 7.67 to 7.21, and Spearman's ρ increases from 0.714 to 0.738. The corresponding differences remain statistically significant after Holm-Bonferroni correction. These results are consistent with the intended use of structured representation learning and explicit task/context modeling under heterogeneous examination conditions. However, the aggregate comparison alone does not isolate which architectural component accounts for the improvement; the contribution of the hierarchical, factorized, sparse, and uncertainty-aware components is examined separately in Section 4.4. On LOCSE, learner-level partitioning ensures that the repeated observations of a given learner remain within a single data partition. HierarchicalFactorizedUncertainty achieves an MAE of 0.451, an RMSE of 0.586, a Spearman's ρ of 0.759, and a QWK of 0.751. Compared with DeBERTa-v3, the proposed method achieves better mean performance for all four metrics, with the reported differences remaining statistically significant after correction. As with SEEFLEX, the overall baseline comparison does not by itself establish whether the improvement arises primarily from hierarchical representation learning, repeated-measurement context, factorized interaction modeling, or uncertainty estimation; these contributions are evaluated through the ablation experiments in Section 4.4. The LOCSE result should therefore be interpreted as improved prediction of observed speaking proficiency across repeated measurement occasions rather than as direct evidence of underlying neural change. HierarchicalFactorizedUncertainty achieves the best mean performance across all metrics reported for the four corpora. The statistical strength of these improvements varies by dataset and metric; in particular, the difference from DeBERTa-v3 in CSEE Spearman's ρ does not remain significant after multiplicity correction. The results therefore support a consistent predictive advantage under the fixed-partition, repeated-run evaluation protocol without implying that every metric-level improvement is statistically significant. Component-specific interpretations are considered in the ablation analysis rather than inferred directly from the aggregate baseline comparison.

### 4.4 Ablation study

To examine the contribution of the major components of HierarchicalFactorizedUncertainty, we conducted a systematic ablation study under the same data partitions, optimization protocol, and repeated-run settings used in the main comparison experiments. Tables 5, 6 report the results on the four English-learning datasets. The ablations were designed to examine the predictive contribution of multi-level hierarchical representation learning, factorized learning-context interaction modeling, sparse regularization, and the epistemic and aleatoric uncertainty mechanisms introduced in Section 3.3. Because separate statistical significance tests are not reported for the ablation comparisons, the discussion below concerns patterns in mean predictive performance across the repeated training runs. Six reduced variants were evaluated. In w/o Hierarchical Composition Mapper, the multi-level attention-based hierarchical stack was replaced by a single projected text representation followed by direct aggregation, while the remaining downstream components were retained. In w/o Factorized Relation Projector, the low-rank interaction between the observation-level latent representation and the recorded task or contextual variables was removed, and the corresponding representations were directly combined before prediction. In w/o Sparse Regularization, the factorized interaction structure was retained but the sparse-regularization coefficient was set to λ = 0. The w/o Epistemic Uncertainty variant retained dropout during training but disabled Monte Carlo dropout at inference, resulting in a single deterministic model realization for prediction. In w/o Aleatoric Uncertainty, the observation-dependent predictive-variance branch was removed and the model was trained as a point predictor. W/o Both Uncertainties removed both stochastic inference and observation-dependent variance estimation, yielding a deterministic point-prediction model.

**Table 5:** Ablation study on CSEE and ELLIPSE.

Variant	CSEE				ELLIPSE			
	MAE ↓	RMSE ↓	*ρ* ↑	QWK ↑	MAE ↓	RMSE ↓	*ρ* ↑	QWK ↑
w/o Hierarchical Composition Mapper	1.21 ± 0.04	1.61 ± 0.05	0.824 ± 0.010	0.831 ± 0.009	0.351 ± 0.010	0.478 ± 0.013	0.727 ± 0.011	0.719 ± 0.010
w/o Factorized Relation Projector	1.17 ± 0.04	1.57 ± 0.05	0.833 ± 0.009	0.839 ± 0.008	0.339 ± 0.009	0.463 ± 0.012	0.739 ± 0.010	0.731 ± 0.009
w/o Sparse Regularization	1.14 ± 0.03	1.53 ± 0.04	0.841 ± 0.008	0.845 ± 0.007	0.329 ± 0.009	0.454 ± 0.011	0.747 ± 0.009	0.739 ± 0.009
w/o Epistemic Uncertainty	1.13 ± 0.04	1.52 ± 0.05	0.843 ± 0.008	0.847 ± 0.007	0.332 ± 0.009	0.458 ± 0.012	0.744 ± 0.010	0.736 ± 0.009
w/o Aleatoric Uncertainty	1.15 ± 0.04	1.55 ± 0.05	0.838 ± 0.009	0.843 ± 0.008	0.337 ± 0.010	0.466 ± 0.012	0.738 ± 0.010	0.730 ± 0.010
w/o Both Uncertainties	1.19 ± 0.05	1.59 ± 0.06	0.829 ± 0.010	0.835 ± 0.009	0.345 ± 0.011	0.472 ± 0.014	0.733 ± 0.012	0.724 ± 0.011
**Full model**	**1.10 ± 0.03**	**1.49 ± 0.04**	**0.847 ± 0.007**	**0.852 ± 0.006**	**0.319 ± 0.008**	**0.445 ± 0.010**	**0.756 ± 0.008**	**0.748 ± 0.008**

MAE and RMSE are lower-is-better (↓), whereas Spearman's ρ and QWK are higher-is-better (↑). Results are reported as mean ± standard deviation over 10 repeated training runs. Bolded values indicate the optimal values.

**Table 6:** Ablation study on SEEFLEX and LOCSE.

Variant	SEEFLEX			LOCSE			
	MAE ↓	RMSE ↓	*ρ* ↑	MAE ↓	RMSE ↓	*ρ* ↑	QWK ↑
w/o Hierarchical Composition Mapper	6.04 ± 0.27	8.01 ± 0.34	0.688 ± 0.019	0.512 ± 0.027	0.661 ± 0.034	0.711 ± 0.021	0.703 ± 0.020
w/o Factorized Relation Projector	5.92 ± 0.25	7.86 ± 0.32	0.697 ± 0.018	0.489 ± 0.025	0.632 ± 0.031	0.728 ± 0.019	0.719 ± 0.018
w/o Sparse Regularization	5.62 ± 0.23	7.46 ± 0.29	0.724 ± 0.016	0.468 ± 0.023	0.608 ± 0.029	0.746 ± 0.017	0.738 ± 0.016
w/o Epistemic Uncertainty	5.77 ± 0.25	7.61 ± 0.31	0.709 ± 0.018	0.501 ± 0.026	0.646 ± 0.033	0.719 ± 0.020	0.710 ± 0.019
w/o Aleatoric Uncertainty	5.69 ± 0.24	7.54 ± 0.30	0.716 ± 0.017	0.482 ± 0.024	0.623 ± 0.030	0.734 ± 0.018	0.726 ± 0.017
w/o Both Uncertainties	5.98 ± 0.28	7.93 ± 0.35	0.694 ± 0.020	0.521 ± 0.029	0.671 ± 0.036	0.701 ± 0.022	0.692 ± 0.021
**Full model**	**5.41 ± 0.22**	**7.21 ± 0.28**	**0.738 ± 0.015**	**0.451 ± 0.022**	**0.586 ± 0.028**	**0.759 ± 0.016**	**0.751 ± 0.015**

QWK is reported for LOCSE because its target is represented on an ordered proficiency scale. Results are reported as mean ± standard deviation over 10 repeated training runs. Bolded values indicate the optimal values.

Table 5 shows the ablation results on the two cross-sectional writing corpora. Among the architecture- and regularization-related ablations, removing the Hierarchical Composition Mapper produces the largest deterioration in mean performance on both datasets. On CSEE, MAE increases from 1.10 to 1.21 and QWK decreases from 0.852 to 0.831. On ELLIPSE, MAE increases from 0.319 to 0.351, with corresponding declines in RMSE, Spearman's ρ, and QWK. These patterns indicate that the multi-level hierarchical component contributes predictive information beyond the fixed pretrained text representation used as its input. Removing the Factorized Relation Projector also reduces mean predictive performance across all reported metrics, although the deterioration is smaller than that produced by removing the hierarchical component. On CSEE, MAE increases from 1.10 to 1.17, while on ELLIPSE it increases from 0.319 to 0.339. This pattern supports the predictive value of explicitly modeling compact associations between latent learning representations and recorded prompt or task context. Removing sparse regularization produces a smaller but consistent deterioration, suggesting that the ℓ_1_-regularized low-rank representation provides an additional, comparatively modest predictive benefit beyond the factorized structure itself. The uncertainty-related ablations show a different pattern. On both CSEE and ELLIPSE, removing the aleatoric mechanism produces a slightly larger deterioration than removing the epistemic mechanism. For example, CSEE MAE increases to 1.15 without aleatoric uncertainty and to 1.13 without epistemic uncertainty; on ELLIPSE, the corresponding values are 0.337 and 0.332. Removing both uncertainty mechanisms produces a larger deterioration than removing either one individually on both corpora. These results are consistent with the two implemented uncertainty mechanisms providing complementary predictive benefits, although the ablations should be interpreted in terms of the complete inference and training mechanisms rather than as a pure experimental separation of underlying uncertainty sources.

Table 6 reports the corresponding results on SEEFLEX and LOCSE. The Hierarchical Composition Mapper again produces the largest mean contribution among the architecture- and regularization-related components. On SEEFLEX, removing the hierarchical component increases MAE from 5.41 to 6.04, while removing the Factorized Relation Projector increases it to 5.92. The comparatively substantial degradation associated with removal of the factorized component is consistent with its intended role in representing associations between learner-language representations and heterogeneous task or contextual information in SEEFLEX. The ablation result, however, does not by itself establish that contextual heterogeneity is the sole cause of this difference. For the uncertainty mechanisms, SEEFLEX and LOCSE show the reverse ordering from CSEE and ELLIPSE: removing epistemic uncertainty produces a larger deterioration than removing aleatoric uncertainty. On SEEFLEX, MAE increases from 5.41 to 5.77 without epistemic uncertainty and to 5.69 without aleatoric uncertainty. On LOCSE, disabling epistemic uncertainty increases MAE from 0.451 to 0.501 and reduces QWK from 0.751 to 0.710, whereas removing aleatoric uncertainty produces an MAE of 0.482 and a QWK of 0.726. When both uncertainty mechanisms are removed, LOCSE MAE increases further to 0.521 and QWK decreases to 0.692. The stronger mean deterioration associated with the epistemic ablation on LOCSE is compatible with the comparatively limited number of independent learners available for model estimation, but this interpretation should be treated cautiously because the epistemic ablation also removes stochastic inference and predictive averaging. More generally, the fact that removing both uncertainty mechanisms consistently performs worse than removing either mechanism alone supports the usefulness of retaining both forms of uncertainty-aware prediction within the proposed framework.

The ablation results indicate that the predictive advantage of HierarchicalFactorizedUncertainty is distributed across multiple components. The Hierarchical Composition Mapper produces the largest mean deterioration when removed among the architecture- and regularization-related components across all four datasets. The Factorized Relation Projector provides an additional and consistent predictive contribution, with a comparatively pronounced effect on SEEFLEX. Sparse regularization yields a smaller but consistent mean benefit, while the epistemic and aleatoric uncertainty mechanisms exhibit dataset-dependent contributions and provide their strongest performance when retained jointly. These findings characterize the computational contribution of the proposed model components to prediction of observable English-learning outcomes and should not be interpreted as evidence that the components correspond to separable cognitive, neural, or neuroplastic mechanisms.

### 4.5 Discussion and practical implications

The empirical results highlight several patterns concerning structured representation learning, learning-context interaction modeling, and uncertainty-aware prediction in secondary-school English assessment. Across CSEE, ELLIPSE, SEEFLEX, and LOCSE, HierarchicalFactorizedUncertainty achieves the best mean performance across the reported prediction metrics relative to the statistical, recurrent, and pretrained Transformer baselines considered in this study. As shown by the ablation experiments, this advantage is distributed across multiple components rather than being attributable to a single architectural element. Among the architecture- and regularization-related ablations, removing the Hierarchical Composition Mapper produces the largest mean deterioration across all four datasets. The Factorized Relation Projector also provides a consistent predictive contribution, with a comparatively pronounced effect on SEEFLEX, where observations are associated with heterogeneous curriculum-based tasks and contextual information. The uncertainty-related ablations exhibit a dataset-dependent pattern. On CSEE and ELLIPSE, removing the aleatoric mechanism results in a slightly larger mean deterioration than removing the epistemic mechanism, whereas the reverse ordering is observed on SEEFLEX and LOCSE. In particular, the epistemic ablation produces a comparatively large degradation on LOCSE, which contains only 104 independent learners. These patterns are consistent with the usefulness of different model components depending on the observational structure of the data. However, the uncertainty ablations modify complete training or inference mechanisms and should therefore not be interpreted as a direct empirical separation of underlying sources of educational variability.

#### 4.5.1 Interpretive boundaries of the neuroplasticity-informed framework

The results should be interpreted within the empirical scope established in Sections 3.1 and 3.2. The four corpora provide written or spoken English productions, externally defined performance or proficiency outcomes, recorded task or contextual information, and, in LOCSE, repeated observations of spoken English over time. They do not provide direct measurements of synaptic modification, neural reorganization, or other biological markers of neuroplasticity. Accordingly, the latent representations learned by HierarchicalFactorizedUncertainty are computational representations optimized for prediction and should not be interpreted as neural states or direct estimates of neuroplastic change. Neuroplasticity instead provides a theoretical motivation for viewing learning-related behavior as adaptive, heterogeneous, experience-sensitive, and potentially variable across repeated observations. Within this interpretation, the empirical results show that hierarchical, context-sensitive, and uncertainty-aware computation can be useful for modeling observable English-learning outcomes. Improved predictive performance does not demonstrate that a particular neural mechanism has been identified. Similarly, variation across the eight LOCSE measurement occasions represents longitudinal change in observed speaking performance rather than direct evidence of neural restructuring. A related boundary concerns causal interpretation. The four datasets are observational secondary data rather than randomized instructional interventions. Associations involving $I_{i}^{\left(t\right)}$ and the Factorized Relation Projector therefore represent predictive relationships between learner-language representations, recorded task or contextual conditions, and observed performance. They do not establish that modifying a particular contextual or instructional condition would cause a corresponding change in learner proficiency. The proposed framework is therefore best interpreted as a predictive and potential decision-support model rather than as a causal model of instructional effectiveness.

#### 4.5.2 Potential decision-support applications

Within these boundaries, the combination of predicted performance and predictive uncertainty may provide useful information for teacher review, although the present study does not prospectively evaluate classroom deployment. A low predicted performance score accompanied by relatively low predictive uncertainty represents a different decision situation from a similar score accompanied by high uncertainty. In the former case, the prediction may provide additional evidence for prioritizing the corresponding learner-language sample for closer review. In the latter case, the uncertainty estimate can motivate collection of additional evidence, such as teacher assessment or an additional language task, before a consequential pedagogical decision is made. For writing assessment, CSEE and ELLIPSE illustrate how predicted holistic performance and uncertainty estimates could be used to prioritize individual essays for diagnostic review. An essay receiving a relatively low predicted score with low uncertainty could be examined for language difficulties requiring further teacher attention, whereas a prediction associated with high uncertainty could be flagged for human reassessment rather than acted upon automatically. Because CSEE and ELLIPSE are treated as cross-sectional corpora in the present experiments, these results do not themselves demonstrate longitudinal tracking of individual writing development. SEEFLEX provides a complementary scenario because it contains learner productions associated with heterogeneous curriculum-based tasks and contextual metadata. The Factorized Relation Projector models predictive associations between latent learner-language representations and these recorded task or contextual variables. Where repeated productions from the same learner are available, variation in predicted performance across task conditions could be used as a hypothesis-generating signal for closer examination of task demands and learner performance. Such patterns would still require verification using the original student work and relevant educational context; the model does not determine which instructional action would cause improvement. LOCSE illustrates a longitudinal decision-support scenario. Because repeated speaking observations from the same learner remain within the same data partition, the framework can characterize predicted speaking proficiency across measurement occasions while also representing uncertainty around those predictions. A sustained pattern of relatively low predicted proficiency could motivate closer examination of the learner's spoken productions or additional formative assessment. Conversely, large changes in predicted proficiency accompanied by high epistemic uncertainty should be interpreted cautiously rather than treated immediately as evidence of genuine progress or decline. The practical implication of the neuroplasticity-informed perspective is not that the model identifies neural change or prescribes a biologically determined instructional intervention. Rather, it motivates treating observable learner performance as heterogeneous and potentially experience-sensitive, while using predictive uncertainty to determine when computational evidence is sufficiently stable to support teacher-led adaptation. Several concrete classroom workflows can be considered within this boundary. For writing tasks, a relatively low predicted performance score accompanied by low predictive uncertainty could be used to prioritize an essay for diagnostic teacher review. The teacher would then inspect the original learner text and the relevant scoring criteria to identify the actual source of difficulty, for example a recurrent problem in linguistic accuracy, lexical use, organization, or task fulfillment. Instruction could subsequently focus on one or two teacher-confirmed difficulties through targeted feedback, guided revision, or a short follow-up writing task. Importantly, the model prediction itself does not diagnose the linguistic error; it determines which samples may warrant earlier or closer human review. A different workflow is appropriate when relatively low predicted performance is accompanied by high epistemic uncertainty. In this case, teachers should avoid immediate placement, remediation, or other consequential decisions based on the model output alone. Instead, an additional essay, speaking task, or teacher-rated formative assessment could be collected. Adaptive support would be introduced only after the additional evidence confirms the initial concern. Similarly, high observation-related variability should encourage the use of multiple performance samples rather than treating a single response as a stable estimate of learner proficiency. For learners with repeated productions under different task conditions, as illustrated by SEEFLEX, consistently lower predicted performance under a particular recorded task condition could prompt the teacher to inspect both the task demands and the corresponding learner work. When the pattern is supported by the original evidence, the teacher could provide additional guided practice, worked examples, task-specific scaffolding, or rehearsal on comparable tasks, followed by performance assessment under a new task condition. Such adaptation should be interpreted as a teacher-generated response to an observed predictive pattern rather than evidence that the task condition causally produced the difficulty. For longitudinal speaking observations, as illustrated by LOCSE, a sustained pattern of relatively low predicted proficiency with low uncertainty across multiple occasions could be used to initiate a short cycle of focused speaking practice followed by formative reassessment. The instructional focus should be selected from teacher examination of the learner's actual spoken production rather than inferred solely from the latent representation. Conversely, an abrupt predicted improvement or decline accompanied by high epistemic uncertainty should trigger verification through an additional speaking sample before the teacher changes the learner's instructional pathway. These examples are prospective uses of model outputs rather than experimentally validated instructional interventions. Their practical value would need to be evaluated in future classroom studies in which teachers interact with model predictions and uncertainty information and subsequent educational decisions and learner outcomes are assessed directly. Such studies could examine whether uncertainty-aware decision support improves the appropriateness, consistency, fairness, or efficiency of teacher review.

#### 4.5.3 Fixed hierarchical depth and model flexibility

The current implementation uses a predefined hierarchical depth of *L* = 3. Although this design provides a consistent computational structure across datasets, it assumes that the same number of representation levels is appropriate for observations and context groups with potentially different structural complexity. The present experiments therefore evaluate a fixed-depth hierarchical architecture rather than automatic discovery of an optimal representation depth. A natural extension would be to introduce an adaptive hierarchical mechanism. Let $h_{l,i}^{\left(t\right)}$ denote the *i*-th observation-level representation at hierarchical level *l*, corresponding to the *i*-th row of $H_{l}^{\left(t\right)}$. A gating function of the form


$$\begin{array}{c} g_{l}^{\left(i,t\right)}=\sigma \left(w_{l}^{⊤}h_{l-1,i}^{\left(t\right)}+b_{l}\right) \end{array}$$ (47)

could determine the contribution of representation level *l* for observation (*i, t*). An adaptive representation could then be constructed as


$$\begin{array}{c} {\tilde{h}}_{i}^{\left(t\right)}=\overset{L_{\max}}{\underset{l=1}{\sum }}g_{l}^{\left(i,t\right)}h_{l,i}^{\left(t\right)}, \end{array}$$ (48)

allowing different observations or context groups to place different weights on shallower and deeper computational representations. Alternative extensions include mixture-of-depth architectures, conditional routing among hierarchical modules, or other learned depth-selection procedures. Such mechanisms could preserve the modular organization of the current framework while reducing dependence on a globally fixed hierarchical depth. The present evaluation is also restricted to English-learning settings. Although the modular representation and uncertainty-aware components are not intrinsically tied to a single school subject, the current experiments do not establish transferability to other curricular domains such as mathematics, science, or social studies. Cross-subject application would require subject-specific input representations, contextual definitions, and independent empirical validation. Accordingly, the present framework should be interpreted as a predictive model of the observed English-learning evidence available in the selected corpora rather than as a complete simulator of classroom dynamics across subjects.

#### 4.5.4 Scope of observed contextual variables

A second limitation concerns the information represented in the selected corpora. English-learning outcomes may be associated with factors beyond learner-language production and recorded task context, including motivation, language anxiety, perceived stress, peer relationships, family background, socioeconomic conditions, and broader educational environment. Some of the selected corpora provide limited demographic, social, or auxiliary cognitive metadata, but these variables are used primarily for sample characterization or sensitivity analysis and are not included in the default predictive feature set. The current framework therefore models only the learner-language, performance-related, task, and contextual information explicitly included in the empirical analysis. Future work could incorporate socio-emotional measurements when they are collected explicitly using appropriate instruments and ethical procedures. Let $s_{i}^{\left(t\right)}$ denote an observed socio-emotional vector containing measurements such as learning anxiety, perceived stress, motivation, or classroom belonging. A straightforward extension would augment the contextual representation as


$$\begin{array}{c} {\tilde{I}}_{i}^{\left(t\right)}=\left[I_{i}^{\left(t\right)};s_{i}^{\left(t\right)}\right]. \end{array}$$ (49)

A more structured alternative would introduce a separate low-rank interaction


$$\begin{array}{c} r_{s,i}^{\left(t\right)}=\left(U_{s}^{⊤}h_{i}^{\left(t\right)}\right)⊙\left(V_{s}^{⊤}s_{i}^{\left(t\right)}\right), \end{array}$$ (50)

thereby distinguishing learning-context associations from learning-socio-emotional associations before fusion. Such an extension could broaden the contextual information represented by the framework while retaining its factorized structure. Sensitive characteristics should, however, be incorporated only when justified by the research question, collected under appropriate ethical procedures, and evaluated for potential privacy and fairness implications. Characteristics that are not explicitly observed should not be inferred from learner-language data. The findings support HierarchicalFactorizedUncertainty as a structured predictive framework for observable English-learning outcomes across heterogeneous cross-sectional and longitudinal corpora. Its potential practical value lies in combining performance prediction, contextual representation, and explicit predictive-uncertainty information. At the same time, the model remains constrained by the variables available in the underlying datasets, the fixed hierarchical architecture, the observational design, and the absence of prospective classroom validation. These limitations should remain explicit when translating model outputs into educational interpretations or future decision-support applications.

## 5 Conclusions and future work

This study introduced HierarchicalFactorizedUncertainty, a neuroplasticity-informed computational framework for modeling heterogeneous English-learning outcomes in secondary-school educational settings. Rather than treating neuroplasticity as a directly observed neural quantity, the framework models observable learner-language and performance-related evidence together with recorded task or contextual information and, where available, repeated learner observations. Multi-level hierarchical representation learning, sparse low-rank learning-context interaction modeling, and aleatoric-epistemic uncertainty modeling are integrated within a unified predictive framework. The empirical evaluation on CSEE, ELLIPSE, SEEFLEX, and LOCSE showed that HierarchicalFactorizedUncertainty achieved the best mean performance across all reported prediction metrics relative to the conventional statistical, recurrent, and pretrained Transformer baselines considered in this study, although the statistical strength of the improvements varied by dataset and metric. The ablation results further indicated that the predictive advantage was distributed across multiple components rather than attributable to a single architectural element. Among the architecture- and regularization-related ablations, removing the Hierarchical Composition Mapper produced the largest mean deterioration across all four datasets. The Factorized Relation Projector provided an additional consistent predictive contribution, with a comparatively pronounced effect on SEEFLEX. The implemented aleatoric and epistemic uncertainty mechanisms exhibited dataset-dependent contributions, and retaining both produced the strongest overall predictive performance among the uncertainty-related variants. These results support the usefulness of structured, context-sensitive, and uncertainty-aware modeling for observable English-learning outcomes.

The findings should nevertheless be interpreted within several important boundaries. The datasets used in this study do not provide direct evidence of synaptic modification, neural reorganization, or other biological measures of neuroplasticity. The learned latent representations therefore constitute computational descriptions optimized for prediction rather than neural states or direct estimates of neuroplastic change. The empirical datasets are observational secondary data. Relationships among learner-language representations, recorded task or contextual variables, and performance should consequently be interpreted as predictive associations rather than causal effects of instructional strategies. The present framework may provide useful information for educational interpretation and future decision-support applications, but it does not establish that modifying a particular instructional or contextual condition will cause a corresponding change in learner proficiency. The current implementation also relies on a predefined hierarchical depth, which may limit flexibility across observations and context groups with different structural complexity. Future research could investigate adaptive hierarchical architectures, including conditional routing, learned layer weighting, and variable-depth representations that allow effective representation depth to vary according to the observed context. In addition, the present model uses only the learner-language, performance-related, task, and contextual variables explicitly included in the selected datasets. Future extensions could incorporate explicitly measured socio-emotional factors such as learning anxiety, perceived stress, motivation, or classroom belonging through additional contextual or factorized representation branches. Appropriately collected family or socioeconomic information could also be considered when justified by the research question. Such extensions should be accompanied by careful privacy, fairness, and missing-data analyses, and characteristics that are not directly observed should not be inferred from learner-language data.

A further direction is prospective classroom validation. The practical scenarios discussed in Section 4.5, such as uncertainty-aware prioritization of writing samples for teacher review or longitudinal examination of predicted speaking proficiency, represent potential decision-support applications rather than experimentally validated interventions. Future studies should therefore evaluate the framework in prospective or quasi-experimental educational settings in which teachers interact with model predictions and uncertainty information and subsequent educational decisions and learner outcomes are assessed independently. Such studies could determine whether uncertainty-aware decision support improves the appropriateness, consistency, fairness, or efficiency of teacher review and whether any resulting changes are associated with improved learner outcomes. Research seeking to make direct claims about neuroplastic change should additionally combine educational observations with appropriate longitudinal neural or physiological measurements, such as neuroimaging or electrophysiological evidence, rather than inferring neural restructuring from behavioral language data alone. This study shows that a neuroplasticity-informed perspective on adaptability, heterogeneity, and experience-sensitive learning can motivate structured computational modeling of English-learning data when combined with explicit treatment of predictive uncertainty. HierarchicalFactorizedUncertainty provides a unified basis for prediction across written, spoken, cross-sectional, and longitudinal English-learning settings while making explicit the uncertainty and interpretive boundaries that should accompany future educational applications.

## Data Availability

The original contributions presented in the study are included in the article/supplementary material, further inquiries can be directed to the corresponding author.

## References

[B1] Abe M. Kondo Y. (2019). Constructing a longitudinal learner corpus to track l2 spoken english. J. Mod. Lang. 29, 23–44. doi: 10.22452/jml.vol29no1.2

[B2] Alfredo R. Echeverria V. Jin Y. Yan L. Swiecki Z. Gašević D. . (2024). Human-centred learning analytics and ai in education: a systematic literature review. Comput. Educ. Artif. Intell. 6:100215. doi: 10.1016/j.caeai.2024.100215

[B3] Alghamdi A. H. (2026). Neuroplasticity in adult second language learning: a systematic scoping review of structural and functional neuroimaging evidence. Front. Hum. Neurosci. 20:1824559. doi: 10.3389/fnhum.2026.1824559 PMC13484637 42614766

[B4] Baker A. E. Galván A. Fuligni A. J. (2025). The connecting brain in context: how adolescent plasticity supports learning and development. Dev. Cogn. Neurosci. 71:101486. doi: 10.1016/j.dcn.2024.101486 PMC11653146 39631105

[B5] Boujmiraz S. Darhmaoui H. Drissi El Maliani A. (2026). Predicting student performance: a comprehensive review of machine learning, deep learning, and explainable AI approaches. Comput. Educ. Artif. Intell. 10:100548. doi: 10.1016/j.caeai.2026.100548

[B6] Crossley S. Tian Y. Baffour P. Franklin A. Kim Y. Morris W. . (2023). The english language learner insight, proficiency and skills evaluation (ellipse) corpus. Int. J. Learn. Corpus Res. 9, 248–269. doi: 10.1075/ijlcr.22026.cro

[B7] de Jong N. H. (2023). Assessing second language speaking proficiency. Annu. Rev. Linguist. 9, 541–560. doi: 10.1146/annurev-linguistics-030521-052114

[B8] Devlin J. Chang M.-W. Lee K. Toutanova K. (2019). “BERT: pre-training of deep bidirectional transformers for language understanding,” in Proceedings of the 2019 Conference of the North American Chapter of the Association for Computational Linguistics: Human Language Technologies, vol. 1 (Minneapolis: Association for Computational Linguistics), 4171–4186. doi: 10.18653/v1/N19-1423

[B9] Drucker H. Burges C. J. C. Kaufman L. Smola A. J. Vapnik V. (1997). “Support vector regression machines,” in Proceeding of Advances in Neural Information Processing Systems, vol. 9 (Cambridge, MA: MIT Press), 155–161.

[B10] Farrelly M. J. Sinwongsuwat K. (2021). Strategies used and challenges faced by thai EFL teachers when eliciting talk during classroom interactions in high school contexts. SAGE Open 11, 1–19. doi: 10.1177/21582440211050385

[B11] Gan L. Lim J. W. (2026). Teacher support and student engagement in second language education: a systematic review of motivational mechanisms and an integrated framework. Humanit. Soc. Sci. Commun. 13:1431. doi: 10.1057/s41599-026-08594-x

[B12] Gawlikowski J. Tassi C. R. N. Ali M. Lee J. Humt M. Feng J. . (2023). A survey of uncertainty in deep neural networks. Artif. Intell. Rev. 56, 1513–1589. doi: 10.1007/s10462-023-10562-9

[B13] Götz S. Granger S. (2024). Learner corpus research for pedagogical purposes: an overview and some research perspectives. Int. J. Learn. Corp. Res. 10, 1–38. doi: 10.1075/ijlcr.00039.got

[B14] Granger S. (2024). From early to future learner corpus research. Int. J. Learn. Corp. Res. 10, 247–279. doi: 10.1075/ijlcr.00050.gra

[B15] He P. Gao J. Chen W. (2023). “DeBERTaV3: improving DeBERTa using ELECTRA-style pre-training with gradient-disentangled embedding sharing,” in Proceedings of Eleventh International Conference on Learning Representations (Appleton, WI: ICLR), 16.

[B16] Hoerl A. E. Kennard R. W. (1970). Ridge regression: biased estimation for nonorthogonal problems. Technometrics 12, 55–67. doi: 10.1080/00401706.1970.10488634

[B17] Karatay Y. (2024). Automated writing evaluation use in second language classrooms: a research synthesis. System 123:103332. doi: 10.1016/j.system.2024.103332

[B18] Leung K. I. Tremblay P. Molnar M. (2026). A systematic review and meta-analysis of language and cognition in the developing bilingual brain: from infancy to adolescence. Aperture Neuro 6. doi: 10.52294/001c.158540

[B19] Li S. Ng V. (2024). “Automated essay scoring: recent successes and future directions,” in Proceedings of the Thirty-Third International Joint Conference on Artificial Intelligence (New York, NY: ACM), 8114–8122. doi: 10.24963/ijcai.2024/897

[B20] Liu D. Qin J. (2024). The effectiveness of cognitive linguistics-inspired language pedagogies: a systematic review. Mod. Lang. J. 108, 794–814. doi: 10.1111/modl.12959

[B21] Liu Y. Ott M. Goyal N. Du J. Joshi M. Chen D. . (2019). RoBERTa: a robustly optimized BERT pretraining approach. arXiv [preprint]. arXiv:1907.11692.

[B22] Misgna H. On B.-W. Lee I. Choi G. S. (2025). A survey on deep learning-based automated essay scoring and feedback generation. Artif. Intell. Rev. 58:36. doi: 10.1007/s10462-024-11017-5

[B23] Paquot M. (2024). Learner corpus research: a critical appraisal and roadmap for contributing (more) to sla research agendas. Corp. Linguist. Linguist. Theory 20, 567–590. doi: 10.1515/cllt-2024-0014

[B24] Pauls T. (2026). Seeflex: The corpus of secondary english as a foreign language (EFL) exams. Int. J. Learn. Corp. Res. 12, 94–119. doi: 10.1075/ijlcr.24027.pau

[B25] Saito K. Macmillan K. Kachlicka M. Kunihara T. Minematsu N. (2023). Automated assessment of second language comprehensibility: review, training, validation, and generalization studies. Stud. Sec. Lang. Acquisit. 45, 234–263. doi: 10.1017/S0272263122000080

[B26] Smale-Jacobse A. E. Meijer A. Helms-Lorenz M. Maulana R. (2019). Differentiated instruction in secondary education: A systematic review of research evidence. Front. Psychol. 10:2366. doi: 10.3389/fpsyg.2019.02366 PMC6883934 31824362

[B27] Taghipour K. Ng H. T. (2016). “A neural approach to automated essay scoring,” in Proceedings of the 2016 Conference on Empirical Methods in Natural Language Processing (Austin, TX: Association for Computational Linguistics), 1882–1891. doi: 10.18653/v1/D16-1193

[B28] Tao L. Wang G. Zhu M. Cai Q. (2021). Bilingualism and domain-general cognitive functions from a neural perspective: a systematic review. Neurosci. Biobehav. Rev. 125, 264–295. doi: 10.1016/j.neubiorev.2021.02.029 33631315

[B29] Tymofiyeva O. Gaschler R. (2021). Training-induced neural plasticity in youth: a systematic review of structural and functional MRI studies. Front. Hum. Neurosci. 14:497245. doi: 10.3389/fnhum.2020.497245 PMC7848153 33536885

[B30] van Hell J. G. (2023). The neurocognitive underpinnings of second language processing: knowledge gains from the past and future outlook. Lang. Learn. 73, 95–138. doi: 10.1111/lang.12601 PMC10881220 38385120

[B31] Vuogan A. Li S. (2023). Examining the effectiveness of peer feedback in second language writing: a meta-analysis. TESOL Quart. 57, 1115–1138. doi: 10.1002/tesq.3178

[B32] Wilbrecht L. Davidow J. Y. (2024). Goal-directed learning in adolescence: neurocognitive development and contextual influences. Nat. Rev. Neurosci. 25, 176–194. doi: 10.1038/s41583-023-00783-w 38263216

[B33] Xiao C. Ma W. Song Q. Xu S. X. Zhang K. Wang Y. . (2025). “Human-AI collaborative essay scoring: a dual-process framework with LLMs,” in Proceedings of the 15th International Learning Analytics and Knowledge Conference (New York, NY: Association for Computing Machinery), 293–305. doi: 10.1145/3706468.3706507

[B34] Ye X. (2024). A review of classroom environment on student engagement in english as a foreign language learning. Front. Educ. 9:1415829. doi: 10.3389/feduc.2024.1415829

[B35] Yeh C. Rowland C. F. Pereira Soares S. M. (2026). How bilingualism influences language processing in the developing brain: a systematic review of the neurobiological evidence. Dev. Rev. 80:101262. doi: 10.1016/j.dr.2026.101262

[B36] Zhai N. Ma X. (2023). The effectiveness of automated writing evaluation on writing quality: a meta-analysis. J. Educ. Comput. Res. 61, 875–900. doi: 10.1177/07356331221127300

[B37] Zhou P. Shi W. Tian J. Qi Z. Li B. Hao H. . (2016). “Attention-based bidirectional long short-term memory networks for relation classification,” in Proceedings of the 54th Annual Meeting of the Association for Computational Linguistics, vol. 2 (Berlin: Association for Computational Linguistics), 207–212. doi: 10.18653/v1/P16-2034

